# Double‐strand DNA break repair: molecular mechanisms and therapeutic targets

**DOI:** 10.1002/mco2.388

**Published:** 2023-10-05

**Authors:** Jinpeng Tan, Xingyao Sun, Hongling Zhao, Hua Guan, Shanshan Gao, Ping‐Kun Zhou

**Affiliations:** ^1^ Hengyang Medical College University of South China Hengyang Hunan Province China; ^2^ Department of Radiation Biology Beijing Key Laboratory for Radiobiology Beijing Institute of Radiation Medicine Beijing China

**Keywords:** DSB repair, HR, Ku70/80 heterodimer/DNA–PKcs (DNA–PK), MRN complex, NHEJ, Therapeutic targets

## Abstract

Double‐strand break (DSB), a significant DNA damage brought on by ionizing radiation, acts as an initiating signal in tumor radiotherapy, causing cancer cells death. The two primary pathways for DNA DSB repair in mammalian cells are nonhomologous end joining (NHEJ) and homologous recombination (HR), which cooperate and compete with one another to achieve effective repair. The DSB repair mechanism depends on numerous regulatory variables. DSB recognition and the recruitment of DNA repair components, for instance, depend on the MRE11–RAD50–NBS1 (MRN) complex and the Ku70/80 heterodimer/DNA–PKcs (DNA–PK) complex, whose control is crucial in determining the DSB repair pathway choice and efficiency of HR and NHEJ. In‐depth elucidation on the DSB repair pathway's molecular mechanisms has greatly facilitated for creation of repair proteins or pathways‐specific inhibitors to advance precise cancer therapy and boost the effectiveness of cancer radiotherapy. The architectures, roles, molecular processes, and inhibitors of significant target proteins in the DSB repair pathways are reviewed in this article. The strategy and application in cancer therapy are also discussed based on the advancement of inhibitors targeted DSB damage response and repair proteins.

## Graphical abstract

Double‐strand break (DSB) is a critical DNA damage induced by ionizing radiation, and which is an actuating signal triggering cancer cell death in tumor radiotherapy. In mammalian cells, nonhomologous end‐joining (NHEJ) and homologous recombination (HR) are the two main pathways of DNA DSB repair, and both cooperate and compete with each other to promote efficient repair. Many regulatory factors play essential roles in the DSB repair pathway. For example, the MRE11–RAD50–NBS1 (MRN) complex and the Ku70/80 heterodimer/DNA–PKcs (DNA–PK) complex are essential for DSB recognition and recruitment of DNA repair factors, and their regulation plays an important role in the choice of the DSB repair HR and NHEJ pathways. Studies and achievements on the molecular mechanisms of the DSB repair pathway may facilitate the development of proteins‐or pathway‐specific inhibitors and advance targeted precise therapy of cancers, and also improving the efficacy of cancer radiotherapy. Here, we review the structures, functions and molecular mechanisms, and inhibitors of key target proteins in the DSB repair pathways. The strategy and application in cancer therapy are also discussed based on the advancement of inhibitors targeted DSB repair proteins.

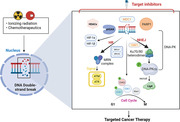

## 1 INTRODUCTION

Deoxyribonucleic acid (DNA) can be harmed by a variety of internal chemicals, external variables like ionizing radiation, and genotoxic chemical stressors. DNA double‐strand breaks (DSBs) are the most serious molecular damaging events that endanger the integrity of the genome and the survival of cells. The two primary methods for DSB repair are homologous recombination (HR) and nonhomologous end joining (NHEJ). Both the NHEJ and HR repair mechanisms depend on two essential protein complexes: the Ku70/80 heterodimer/DNA–PKcs (DNA–PK) complex and the MRE11–RAD50–NBS1 (MRN) complex.^1^, ^2^, ^3^, ^4^, ^5^

**FIGURE 1:**
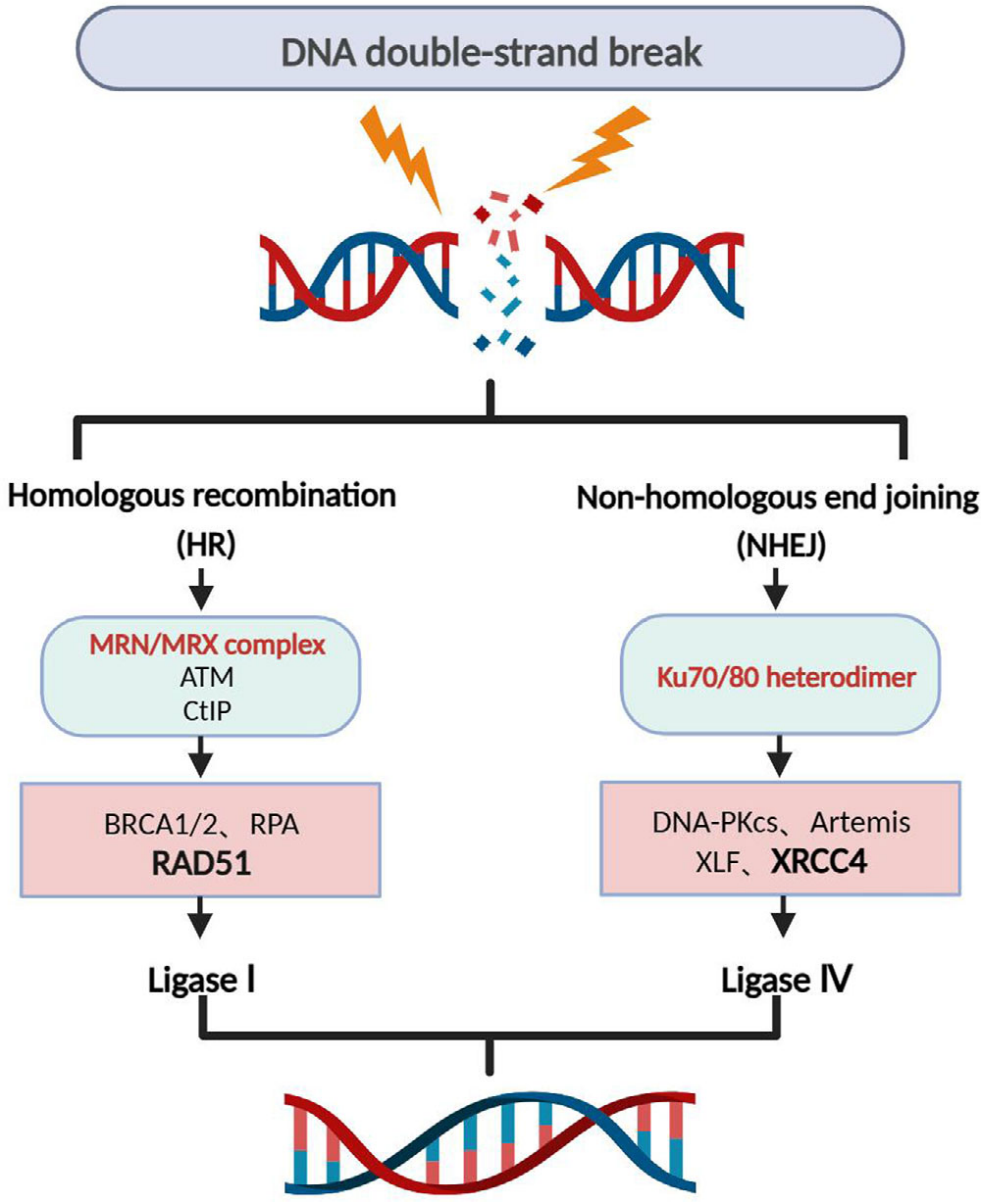
Schematic diagram showing the nonhomologous end joining (NHEJ) and homologous recombination (HR) pathways of DNA double‐strand break (DSB) repair.

A circular protein called the Ku70/80 heterodimer identifies a DSB site to start the NHEJ repair pathway and binds the frayed DNA ends together. It subsequently brings Artemis nuclease and DNA‐dependent protein kinase catalytic subunit (DNA–PKcs) to the DSB site. To join the fragmented DNA ends, DNA ligase IV (Lig4), XRCC4, and XRCC4‐like factor (XLF) are recruited once DNA–PKcs is activated.^4^, ^6^, ^7^, ^8^, ^9^ MRN is the first protein complex in the HR repair pathway to recognize DSB sites and initiate HR pathway. During the S or G2 phase of the cell cycle, when sister chromatids are available as homologous DNA templates, the MRN complex and its cofactor Sae2 form a nuclease complex that excises the single‐stranded ends of DNA DSBs, releasing the 5′ end oligonucleotides and forming a 3′ single‐stranded DNA (ssDNA) bridge.^10^ The ssDNA‐binding protein, replication protein A (RPA), can encapsulate the protruding end. BRCA1 and BRCA2 then promote the exchange of RAD51 with RPA‐encapsulated ssDNA to create RAD51 nuclear filaments. The newly formed ssDNA–RAD51 core filaments then enter the sister chromatid to form the D‐loop and guides DNA synthesis along the ssDNA template^4^, ^11^, ^12^ (Figure 1).

In the molecular mechanisms of DSB repair, there is little discussion of how MRN complexes and Ku70/80 heterodimers compete synergistically in the choice of repair pathways for DNA DSB damage. Therefore, this review may lead to a better understanding and appreciation of the choice regulation of DNA damage repair pathways and therapeutic targets for cancer radiotherapy through the synergistic competition between MRN complexes and Ku70/80 heterodimers as well as therapeutic targets in the DSB repair pathway. This review focuses on the following topics: structure and function of key HR and NHEJ pathway proteins, synergistic competition between MRN complexes and Ku70/80 heterodimers, and target proteins for HR and NHEJ pathway DSB therapies.

## 2 HR AND NHEJ REPAIR PATHWAYS

### 2.1 HR

In many species, including yeast and humans, HR is a tightly controlled molecular mechanism that maintains genome integrity by repairing DSBs that block DNA replication forks. The most important feature of HR is to use homologous sister chromatids to template error‐free DSB repair. When meiosis is occurring normally or following exposure to genotoxic substances or ionizing radiation, HR offers precise DSB repair. The HR pathway is initiated by recognition of the damage site by the MRN complex, which functions in several ways: first, it anchors damaged DNA ends structurally in DSB lesions via RAD50. It then performs end resection of the broken DNA via MRE11 nuclease activity together with BRCA1 to produce 3′‐end ssDNA.^13^, ^14^, ^15^ The ssDNA‐binding protein, RPA, binds directly to the DSB site, removing DNA secondary structure and preparing nucleoprotein filaments for RAD51 formation. In contrast, ATR, via RPA–ATRIP (ATR‐interacting protein) interactions, binds to RPA‐bound ssDNA and engages the ATR–Chk1 DNA damage checkpoint, preventing replication forks from progressing through DNA lesions by inducing cell cycle arrest. NBS1 functions as a substrate for ATM and facilitates the recruitment of ATM and activation of checkpoints. RAD51 is recruited to the site of DSB through binding with BRCA2, which is facilitated by PALB2, the protein that links BRCA1 and BRCA2. Once successfully released from BRCA2, RAD51 forms a nuclear filament that allows entry into the cognate DNA double helix, which HR uses as a template for DNA synthesis and repair.^10^, ^15^, ^16^

HR defects (HRD) are impairments in the HR repair pathway that are related to various tumor types, such as breast, ovarian, prostate, and pancreatic cancers. The HRD phenotype can increase tumor sensitivity to platinum‐based drugs induced by ICL and PARP inhibitors (PARPi).^15^, ^17^ HRD was identified as a predictive biomarker for PARPi in the management of ovarian cancer, according to patient outcomes in a phase III trial with randomized control.^18^, ^19^, ^20^, ^21^ The BRCA1 and BRCA2 genes are significant players within the HR pathway, with the impaired function of these genes being the most studied cancer cellular mechanism relating to HRD. HRD phenotypes in breast, ovarian, pancreatic, and prostate cancers have been associated with germline and somatic mutations as well as suppression of epigenetic modifications in BRCA1 and BRCA2. As a consequence, the genes have been implicated as prototypes for determining HRD phenotypes.^22^ Other HR pathway genes that are associated with the HRD phenotype comprise ATM, PALB2, and RAD51. Epigenetic or genetic modifications, or specific combinations of these genes, are liable for the manifestation of the HRD phenotypes in a variety of cancer types, such as ovarian,^23^, ^24^, ^25^ endometrial,^26^, ^27^, ^28^ breast,^29^, ^30^, ^31^ prostate,^32^, ^33^, ^34^ and pancreatic cancers.^35^, ^36^, ^37^ As we study HR and HRD in greater depth, more therapeutic targets for HR will emerge, as well as related targeted drugs for a wider range of HR‐deficient tumor therapies.

### 2.2 NHEJ

Another important mechanism for DSB repair is NHEJ. The primary mechanism for DSB repair in somatic cells, both proliferating and nondividing, is NHEJ.^4^ Outside of the S and G2 phases of the cell cycle, NHEJ almost always repairs DSBs in human cells. NHEJ may be able to repair up to 80% of the DSBs brought on by ionizing radiation even during the G2 phase. The first factor to bind to DSBs in NHEJ is the Ku70–Ku80 heterodimer. Other NHEJ proteins may then be directly or indirectly recruited by it. To prevent excessive excision of DNA break ends, Ku70–Ku80 heterodimers are abundant in primate cells and have a high affinity for binding to DNA break ends. The XRCC4–DNA ligase 4 (Lig4) complex directly ligated DNA ends, and its activity was boosted by XRCC4‐like factor (XLF) and/or XRCC4 and XLF homologues (PAXX). But frequently, DNA end processing—which can involve nucleotide addition, modification, or excision—is necessary for repair. End processing is reliant on the nuclease activity of Artemis, the kinase activity of DNA–PKcs, the addition of nucleotides by polymerase‐μ (Pol μ) and Pol λ, and the modification of nucleotides by tyrosine‐DNA phosphodiesterase 1 and polynucleotide kinase 3′‐phosphatase.^8^, ^38^, ^39^

Because radiation causes DSBs, defects in NHEJ proteins significantly increase radiosensitivity in cell lines and mouse models. V(D)J recombination, which involves the introduction and reattachment of designed DSBs to produce a variety of T and B cells, contributes to the maturation of the immune response. NHEJ unites these programmed DSBs. Therefore, NHEJ deficiency results in (severe) combined immunodeficiency (S)CID because V(D)J recombination cannot be carried out effectively. Another stage in increasing T and B cell diversity is class‐switch recombination, which is facilitated by NHEJ.^40^

## 3 THE MRN COMPLEX IN HR

The MRN protein complex, made up of MRE11, RAD50, and NBS1, plays a crucial regulatory role in the HR route, boosting the HR pathway repair of DNA DSBs. A significant protein complex that is essential for identifying DNA DSBs and for signaling that results in DSBs repair is the evolutionarily conserved MRN complex.^2^ As a DSB sensor, the MRN complex participates in the initial phase of the DNA damage response (DDR). Ataxia–telangiectasia‐mutated (ATM) signaling pathways can develop abnormalities as a result of mutation, knockout, degradation, or mislocalization of MRN complex components, according to prior research.^41^

The MRN complex is the initiation complex in the HR repair pathway. MRN complexes are rapidly recruited to DSB sites, where they initiate DSB end resections and maintain DSB ends in preparation for subsequent repair. In addition, ATM is recruited and activated to coordinate DSB repair and cell cycle progression.^42^, ^43^, ^44^ ATM kinase activity in cells is initiated by intermolecular autophosphorylation at S1981, S367, and S1893.^43^ The autophosphorylation of ATM enables it to dissociate into active monomer and be reactivated, enabling ATM to remain at DNA damage sites, where it catalyzes necessary downstream phosphorylation events.^41^, ^43^, ^45^ When ATM is activated at DSBs, histone H2AX is phosphorylated to produce H2AX, which then attracts and phosphorylates MDC1 and activates two E3 ubiquitin ligases, RNF8 and RNF168, to start a ubiquitination (UB) cascade.^2^ Chromatin conformation changes caused by a series of signaling cascades promote the recruitment of multiple factors, including BRCA1, CtIP (MRX and Sae2 in yeast), EXO1, and BLM.^46^ The MRN complex and CtIP form complexes with BRCA1, which is essential for facilitating DSB end resection.^47^, ^48^

The MRN complex is at the heart of an intricate network that detects damaged DNA and eventually encourages its repair by activating signaling pathways. In particular, the activation of the signal transduction cascade passing through the cell cycle checkpoints is greatly aided by the DSB sensor, MRN complex. Additionally, it is essential for controlling the choice of repair pathways and the DNA repair procedure carried out by NHEJ and HR. Understanding this complex's operations will help clarify the conditions necessary to sustain genomic integrity.^41^

### 3.1 MRE11

#### 3.1.1 Structure of MRE11

**FIGURE 2:**
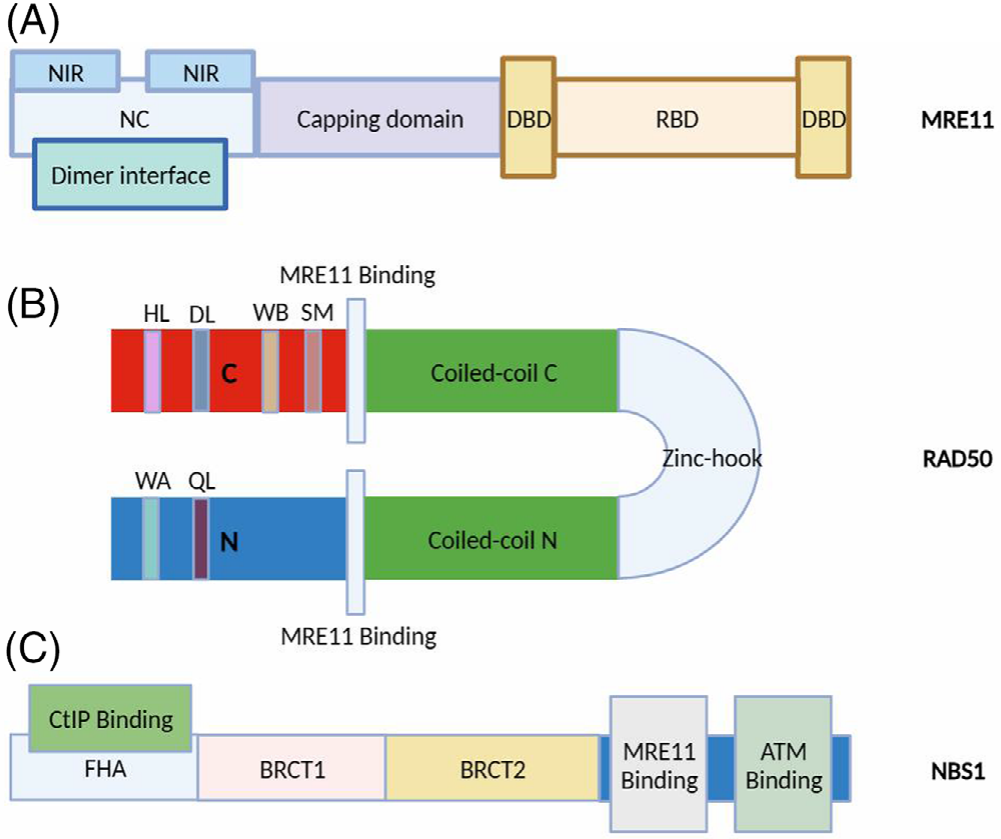
MRE11, RAD50, and NBS1 schematic structures. (A) Structure of MRE11. The protein comprises two DNA‐binding domains (DBDs), one in the N‐terminus of the RAD50‐binding domain (RBD), and the other in the C‐terminus. The N‐terminal nuclease core (NC) domain of the protein contains an NBS1‐interacting region (NIR). (B) Structure of RAD50. Walker A (WA) and Q‐loop (QL) are found on the N‐terminal end of RAD50, and Signature Motif (SM), Walker B (WB), D‐loop (DL), and H‐loop (HL) are found on the C‐terminal end. RAD50 forms dimers through zinc ion‐containing hook domains. Additionally, it has MRE11 binding regions at both its C‐ and N‐termini. (C) Structure of NBS1. The N‐terminal end of the NBS1 structure is equipped with the BRCT structural domain and the FHA structural domain (containing the CtIP‐binding region), while the C‐terminal end of the structure has the ATM‐binding region and the MRE11‐binding region.

The *Mre11* gene in humans has 20 exons, is found on chromosome 11, and codes for a protein of 708 amino acids (aa). The N‐terminal core domain of the highly conserved 70−90 kD protein MRE11 is what allows it to dimerize.^49^ The MRE11 protein is made up of two DNA‐binding domains (DBDs), one at the N‐terminus of the RAD50‐binding domain and the other at the C‐terminus of the nuclease core domain (NC domain), which contains an NBS1‐interacting region (NIR). The U‐shaped pocket‐like dimer that human MRE11 generates allows it to bind various DSBs either symmetrically or asymmetrically. MRE11 can bind DNA after dimerization and is essential for DSB repair.^50^ In *Saccharomyces cerevisiae*, the N‐terminal domain of MRE11 affects the excision activity of remote nucleases by mediating the association of MRX and Tel1/ATM with DNA DSBs, while the C‐terminal domain (CTD) promotes MRX‐tethering activity by interacting with RAD50^51^ (Figure 2A).

#### 3.1.2 Function and modification of MRE11

**FIGURE 3:**
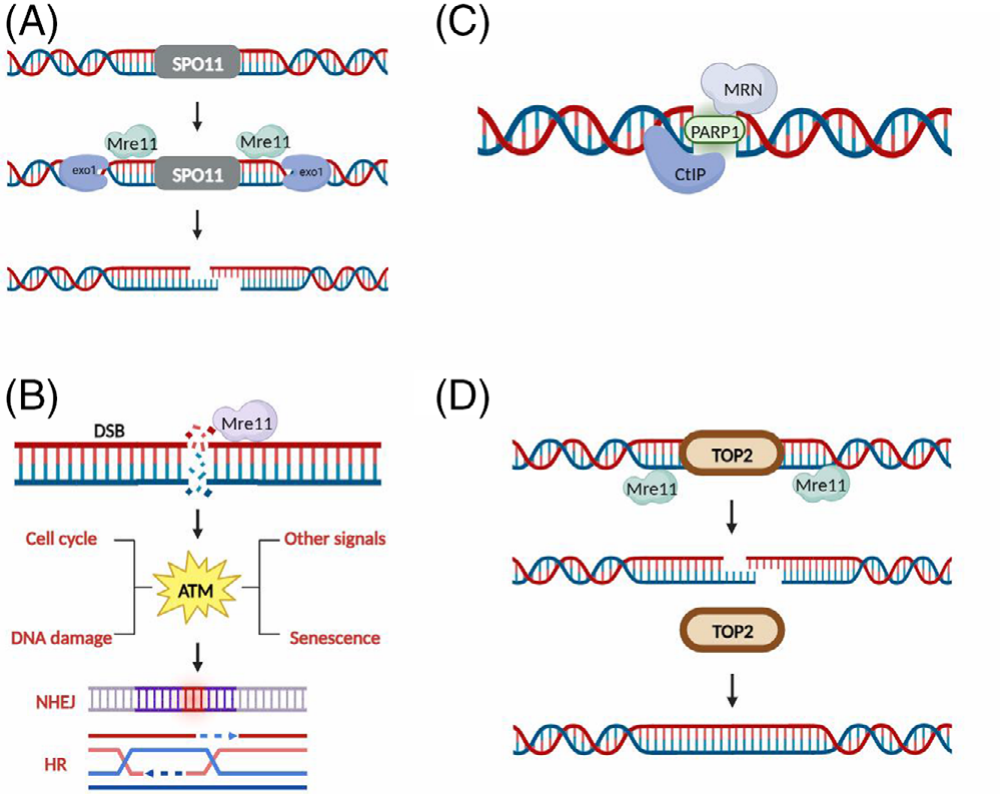
Biological functions of MRE11. (A) MRE11 removes Spo11, which shows endonuclease activity and is involved in meiosis. (B) MRE11 is a component of the overall DNA damage response (DDR), which includes DSB identification, signal transduction, and repair. (C) MRE11 participates in V(D)J recombination as well. (D) MRE11 removes toxic DNA adducts.

MRE11 is an essential component of the MRN complex. Recombinant MRE11 shows high 3′→5′ exonuclease activity.^46^, ^52^, ^53^ The MRE11 protein is involved in meiosis,^54^, ^55^ DSB repair,^56^, ^57^ V(D)J recombination,^58^, ^59^ telomere maintenance,^60^, ^61^, ^62^ and adduct removal (Figure 3). MRE11 commonly functions with other proteins, for example, MRE11 cuts DSB ends with topoisomerase II, or in combination with EXO1, DSBs are resected bidirectionally to remove the topoisomerase‐like protein Spo11.^50^, ^63^ After DNA is damaged, MRE11 activates the ATM‐ or ATR‐mediated DDR, regulates important steps in repairing damaged DNA, and plays an important role in processing broken DNA ends.^50^

**FIGURE 4:**
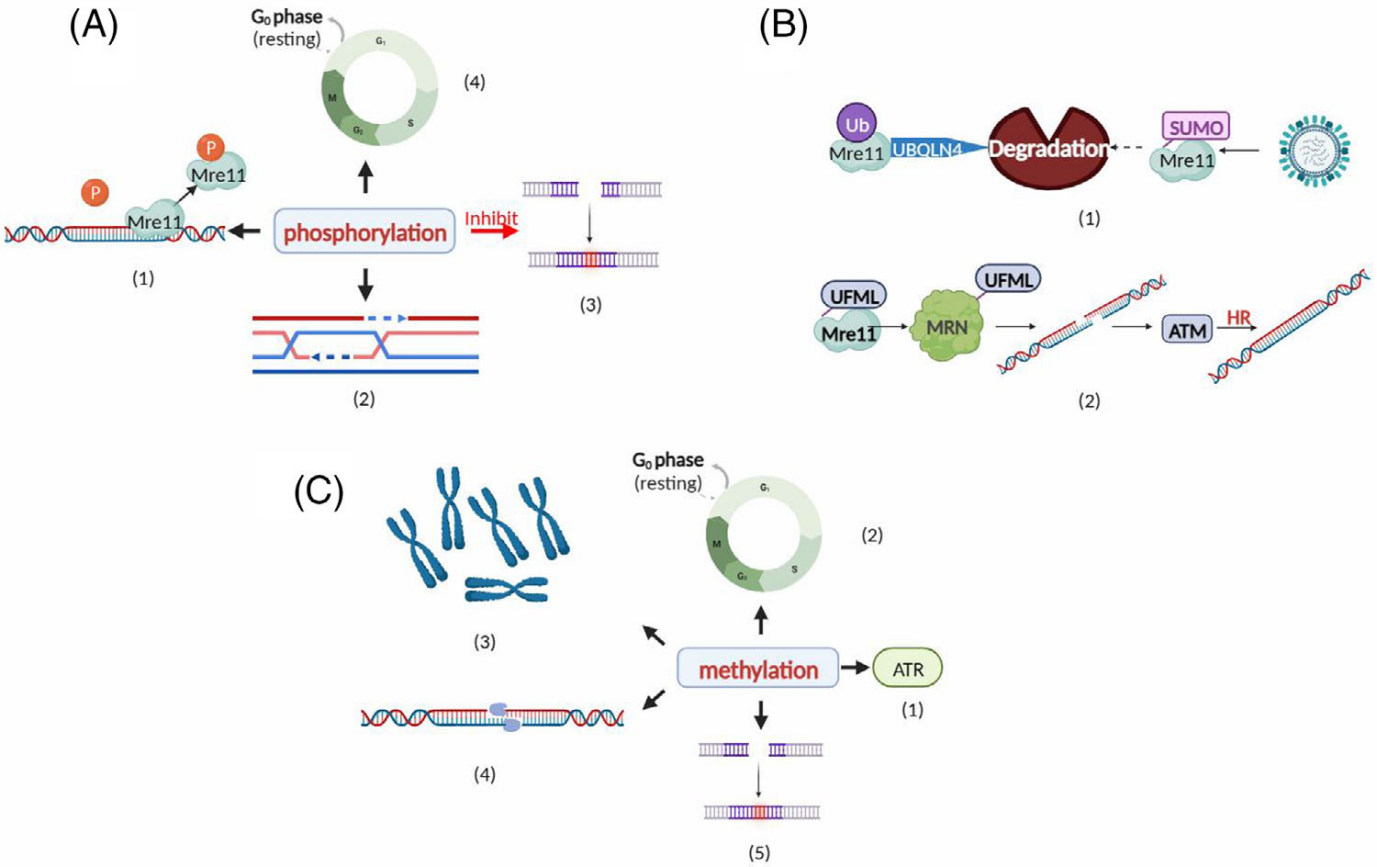
Relevant biological roles played by posttranslational modifications (PTMs) on MRE11. (A) Effects of MRE11 phosphorylation: (1) enhanced affinity for DNA; (2) the encouragement of the HR repair; (3) NHEJ repair blockage; and (4) regulation of cell cycle checkpoints. (B) Effects of MRE11 modification via ubiquitination (UB) and ubiquitin‐like modification (ULB). (1) Effects of MRE11 UB and SUMOylation: SUMO‐MRE11 is targeted by an adenovirus, but MRE11 UB enhances its UBQLN4‐mediated destruction. (2) MRE11's function in deacylation is to facilitate the development of the MRN complex and DNA repair. homologous recombination, or HR. (C) Effect of methylation on MRE11: (1) regulation of cell cycle checkpoints; (2) promotion of ATR signaling; (3) promotion of alt‐NHEJ repair; (4) regulation of exonuclease activity; and (5) maintenance of normal chromosome morphology.

A growing body of research indicates that MRE11 undergoes a variety of posttranslational modifications (PTMs), and that these PTMs confer or enhance MRE11's regulatory activities in numerous biological processes, such as DNA damage recognition, DNA binding, nuclease activity, and signaling.^50^ Thirty‐eight sites in MRE11 were identified to be phosphorylated, and 14 of those sites have obvious biological activities, according to information in the *PhosphoSitePlus* database. MRE11 phosphorylation can change MRE11's ability to bind DNA, control whether HR and NHEJ repair pathways are used, and have an impact on the cell cycle and chromosome alignment.^50^ It has been demonstrated that PI3K‐related kinase (PIKK) can phosphorylate the SQ/TQ motif in MRE11 to cause the MRN complex to separate from chromatin by decreasing MRE11's affinity for DNA.^64^ MRE11 has also been reported to be phosphorylated by ATM at S676 and S678 in response to a reagent that induces DNA DSBs, and this modification depended on the presence of NBS1 and did not affect the association of MRN complex members or ATM activation. This study describes a new role of ATM‐dependent MRE11 phosphorylation: limiting the extent of excision mediated by exonase 1.^65^ In summary, MRE11 shows a reduced affinity for DNA after phosphorylation and participates in many cellular activities and processes (Figure 4A).

UB is the other most common PTM of MRE11. Ubiquitinated MRE11 can be recognized and degraded by the proteasome. A different PTM called SUMOylation, which includes the addition of a small ubiquitin‐like modifier (SUMO), controls the subcellular location of MRE11. Additionally, the presence of that ubiquitin‐fold modifier protein (UFMylation) regulates the assembly of the MRN complex and preserves telomere length rather than causing the destruction or stabilization of the MRE11 protein.^50^ MRE11 is UFMylated at K282, a modified residue that is required for MRN complex formation, optimal DSB‐induced ATM activation, and HR‐mediated repair and genome integrity under physiological conditions.^66^ MRE11 requires SUMO acidification to protect it from ubiquitin‐mediated degradation during the excision of damaged chromatin. After DSBs are introduced, PIAS1 promotes the SUMO acidification of MRE11 on chromatin to acidify the protein, which then initiates DNA end excision. Then, MRE11 detaches from chromatin and is deSUMOylated by SENP3. Therefore, SENP3 deficiency leads to failed MRE11 degradation and its accumulation on chromatin, causing genomic instability. This study further demonstrated that tumor‐associated MRE11 mutants with impaired SUMO acidification exhibited impairment of the DNA repair capacity. Hence, this study demonstrated that MRE11 SUMOylation, in tandem with UB, is dynamically controlled by PIAS1 and SENP3 to facilitate DNA end excision and maintain genomic stability.^67^ However, in contrast to studies of MRE11 phosphorylation, further in‐depth studies into MRE11 UB and ubiquitin‐like modifications (UBLs) are urgently needed (Figure 4B).

MRE11 is the first one to be identified as an arginine‐methylated protein in an early proteomic study in which arginine methyl‐specific antibodies were used. The literature shows that MRE11 can be targeted to damaged DNA foci via methylated GAR motifs and colocalizes with γH2AX, which facilitates DSB sensing and DDR initiation. In addition, the methylation of human MRE11 promotes alt‐NHEJ, an NHEJ subpathway. These discoveries suggest a new direction for the study of the mechanism by which MRE11 precisely regulates DSB repair by mediating the alt‐NHEJ subpathway. Invertebrates, vertebrates, and microbes all exhibit MRE11 methylation under physiological and stressful conditions, and this PTM is crucial for preserving cellular genomic stability.^50^, ^68^ Studies have shown that methylation regulates MRE11's association with nuclear structures, such as PML nuclear bodies and DNA damage sites. Through cell fractionation assays, a study proved that MRE11 methylation was mainly related to nuclear structure, and MRE11 methylation at an arginine residue was necessary for this association.^68^ A mutation in MRE11's methylation arginine residue significantly decreased the protein's activity with ribozymes but did not affect its capacity to bind to RAD50 and NBS1. Hypomethylated MRE11‐containing cells displayed abnormalities in the S‐phase DNA damage checkpoint that were mostly repaired by the MRN complex. The MRN complex's activity in the S‐phase DNA damage checkpoint response is thus regulated by arginine methylation, and MRE11's arginine methylation is required for DNA damage checkpoint control^69^ (Figure 4C).

Following DSB introduction, the role of MRE11 as a defensive player is regulated by multiple PTMs. However, except for phosphorylation, UB, and methylation, we know little about the other PTMs of MRE11. The majority of MRE11 PTMs seem to have significant and varied functions in DNA repair pathway selection and signaling. In contrast, methylation and UB enhance alt‐NHEJ and, respectively, NHEJ repair. As an illustration, phosphorylation of MRE11 particularly blocks NHEJ repair. Different PTMs regulate MRE11 dynamically, changing the cell's fate.^50^

### 3.2 RAD50

#### 3.2.1 Structure of RAD50

The human enzyme RAD50 comprises 1312 aa and is the largest component in the MRN complex.^49^ The ATP binding cassette (ABC) protein superfamily member RAD50 possessed three P‐loop nucleoside triphosphate hydrolase (P‐loop NTPase) domains.^70^ RAD50 also forms a highly conserved zinc (2+)‐dependent homodimer, mediated primarily by its hook domain. Studies have shown that the mouse RAD50 hook domain profoundly influences MRN complex‐dependent DDR signaling, tissue homeostasis, and tumorigenesis.^71^ RAD50 includes six distinct motifs, including Walker B, D‐loop, and H‐loop/switch domains at the C‐terminus and an N‐terminal Walker A and Q‐loop. This information was obtained from the residue annotation of the protein.^70^ The RAD50 protein harbors a globular ABC ATPase domain called the head. One zinc ion in the coordination complex interacts homologically with four RAD50 cysteine residues (two on each monomer) via the zinc hook (not the SMC hinge domain). The zinc hook and head form an extended coil ‘arm’ with distant connections that undergo coordinated structural transitions during the ATPase cycle, which regulates enzymatic functions and free energy levels.^72^ In the hook domain of *Pyrococcus furiosus* RAD50 (PfRAD50), the proximal helix of each RAD50 protomer is oriented 140 degrees from the respective globular domain, a conformation driven by Zn2+ synergy.^73^ It has been established that RAD50 endonuclease activity and the activation of the ATM kinase depend on the coordinated hydrolysis of two ATPase sites in the RAD50 ATP‐binding dimer^74^ (Figure 2B).

#### 3.2.2 Function and modification of RAD50

RAD50 binds to DSB sites early in the repair process, coordinating their close juxtaposition and activating the ATM kinase critical for DNA damage signaling.^70^ The breast cancer susceptibility gene RAD50 encodes a protein that is crucial to the DDR for preserving genomic integrity and inhibiting tumor development.

At S635, RAD50 is phosphorylated by ATM, activating it to participate in DNA repair and cell cycle checkpoints. RAD50 plays a crucial part in the ATM‐dependent DDR pathway in addition to serving as a tumor suppressor protein.^75^, ^76^ DNA repair as well as cell cycle control are regulated by the RAD50 protein's S635 site, which is phosphorylated by the ATM protein.^77^ Recent research has demonstrated that the duration of mitosis is influenced by RAD50. RAD50‐deficient fibroblasts showed a substantial delay in mitotic progression, which may be corrected by the lentiviral transduction of RAD50.^78^ Purified human RAD50 demonstrates ATPase activity, ATP‐dependent conformational changes, and ATP binding, according to studies. RAD50 binds DNA, not oligomers, as demonstrated by scanning force microscopy study, although RAD50 alone is not bound to DNA molecules. RAD50 multimers are spherical and do not have helical coil extensions; in contrast, MRE11–RAD50 (MR) in complex exhibits significant helical coils protruding from the center region after ATP‐induced oligomerization. The structural arrangement of RAD50 is stabilized and maintained by MRE11 in the ATP‐bound state.^79^ Mutations in the RAD50 hinge region also significantly increase its ATP hydrolysis activity via its characteristic ABC ATPase motifs. An increase in MRE11 exonuclease activity in a presence of ATP is closely correlated with changes in RAD50 ATPase activity.^80^ Additionally, the global alterations in the MRE11–RAD50 structure brought about by the binding of ATP to RAD50 are crucial for the performance of DNA repair. In this study, a number of mutants were subjected to methyl‐NMR spectroscopy, and the results were used to characterize the dynamic allosteric pathway mediated by RAD50. The results of the experiments showed a balance between RAD50's ground state and active dimeric state, whose structure and kinetics are changed during ATP‐induced dimerization and ultimately allow for ATP hydrolysis. For DNA DSB repair that is controlled by MRE11–RAD50, this sparse intermediate is necessary.^81^ The RAD50 mutant S793R's crystal structure has been published. A missense mutation in the characteristic motif changes key serine residues in the RAD50 homologue and the conserved characteristic motif in the ABCATP enzymatic domain. A serine‐to‐arginine change in the RAD50 protein specifically hindered ATP binding and interfered with communication between other ATP‐binding loops, according to structural analyses. Due to the changed communication between RAD50 monomers as a result of this structural modification, RAD50 dimerization was prevented. A mutant protein that formed complexes with MRE11 and NBS1 was produced by an analogous mutation in the human RAD50 gene, but all ATP‐dependent enzymatic activity was lost.^82^

**FIGURE 5:**
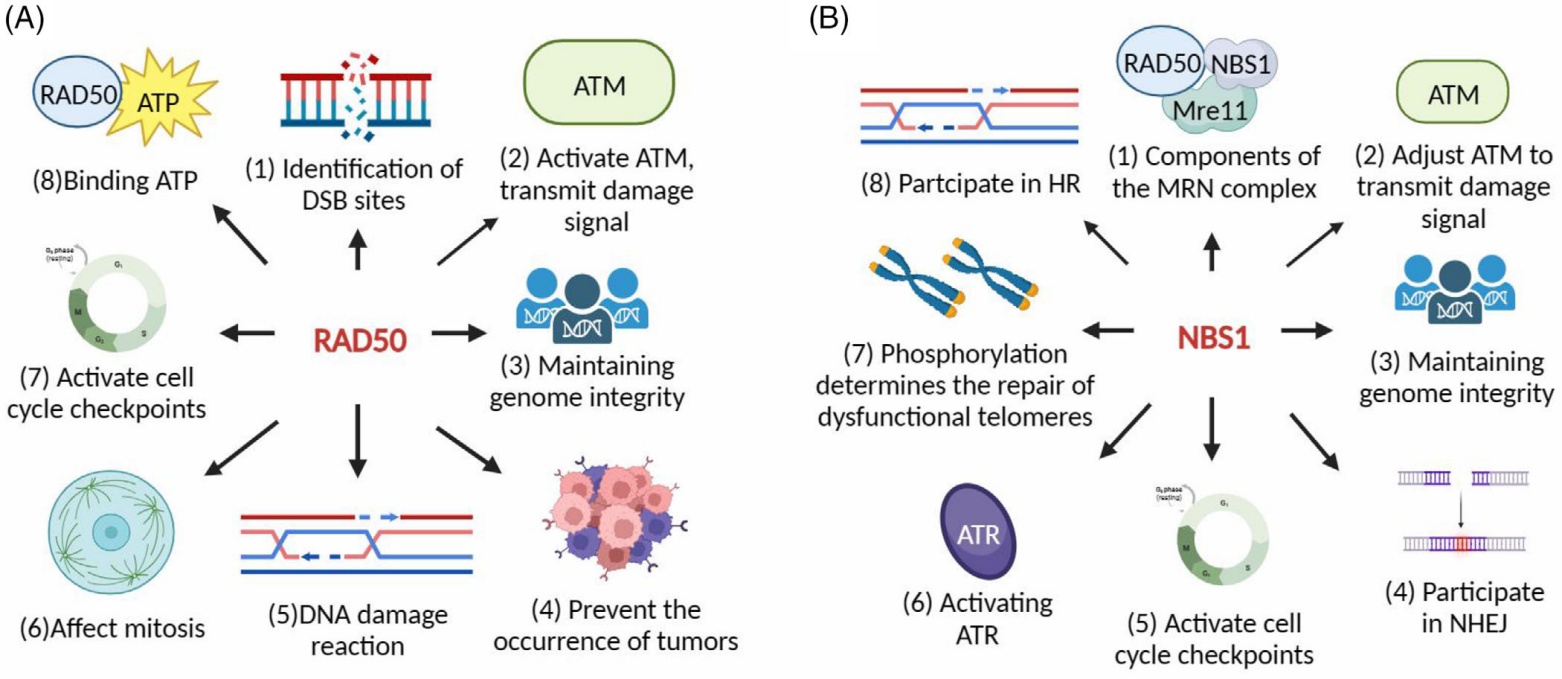
Biological function and modification of RAD50 and NBS1. (A) (1) Rad50 identifies DSB sites; (2) activates ATM kinase, which transmits DNA damage signals downstream; (3) maintains genome integrity; (4) prevents the development of tumors; (5) participates in the DDR after being methylation; (6) impacts the length of mitosis; (7) activates cell cycle checkpoints after ATM phosphorylation of S635; and (8) binds ATP, undergoes ATP‐dependent conformational changes, and exhibits ATPase activity. (B) (1) A vital part of the MRN complex is NBS1. (2) Additionally, NBS1 controls the ATM kinase, which broadcasts signs of DNA damage. Skp2 ubiquitinates the C‐terminus of NBS1 (lsi‐735 and lsi‐751 sites), which encourages ATM activation. Similar to this, the interaction between the NBS1 BRCT domains and poly (ADP‐ribose) is crucial for early ATM activation. (3) NBS1 protects genome integrity. (4) By altering RNF20‐dependent H2B UB, H2A/H2AX ubiquitination, and H2A/H2AX phosphorylation, NBS1 may contribute to NHEJ repair. (5) NBS1 activates cell cycle checkpoints. (6) NBS1 promotes ATR activation through its interaction with RPA32 and TopBP1, and the N‐terminal region of NBS1 can directly activate ATR independent of its interaction with TOPBP1. (7) Based on telomere dysfunction, the repair pathway is chosen according to NBS1 phosphorylation state. In the S/G2 phase, for instance, CDK2's phosphorylation of S432 in NBS1 isolates NBS1 from TRF2, encourages the assembly of the TRF2 Apollo/SNM1B complex, and safeguards the leading‐strand telomere. (8) NBS1 may interact with nucleolar proteins by interacting with γ‐H2AX, thus playing a role in clearing damaged H2A/H2B nucleosomes and promoting homologous recombination (HR) repair.

Additionally, it has been demonstrated that methylated residues in RAD50 react to DNA damage brought on by ‐rays. Walker B aspartic acid and glutamate residue methylation is connected to ATP hydrolysis, and the posttranslational methylation is particularly important in controlling the paleontological MR complex and DDR.^83^ After DSB occurrence, RAD50 is regulated by multiple PTMs. However, we know little about RAD50 PTMs except those involving phosphorylation and methylation. Therefore, more in‐depth research is needed on other PTMs of RAD50 to better understand all of the diverse biological roles of RAD50 in DNA repair pathway choice and signaling, cell proliferation, and so on (Figure 5A).

### 3.3 NBS1

#### 3.3.1 Structure of NBS1

In mammalian cells, NBS1 is a component of the MRN complex.^84^, ^85^ N‐terminal domain (aa 1−196), core domain (aa 278−343), and CTD (aa 665−693) are the three functional domains that make up the NBS1 protein.^43^, ^85^

NBS1's N‐terminal FHA and BRCT domains are necessary for the protein to bind to phosphorylated proteins found in ionizing radiation‐induced nuclear foci (IRIF), including H2AX, the mediator of CtBP‐interacting protein (CtIP), and DNA damage checkpoint protein 1 (MDC1).^85^, ^86^, ^87^, ^88^ The highly conserved and essential domain found at the C‐terminus of NBS1 is involved in DSB repair signaling. Although it is believed that this domain lacks any discernible structural folds, it does have an expanded structure that facilitates the recruitment and activation of ATM/Tel1.^87^ The N‐terminal forkhead‐related domain, not the important residues involved in ATM phosphorylation (S278 and S343), nor the evolutionarily conserved C‐terminal region (CTR) of NBS1, is required for survival after G2/M and S‐checkpoint arrest and radiation‐induced DNA damage in the development of T cells and oocytes. However, CTR is involved in regulating radiation‐induced apoptosis.^89^ The essential characteristics of NBS1 were revealed in a study that used X‐ray crystallography and small‐angle X‐ray scattering to characterize NBS1 structures in fission yeast and humans. These features included a fused, extended FHA–BRCT1–BRCT2 domain that was flexible linked to the C‐terminal MRE11‐ and ATM‐binding motif^90^ (Figure 2C).

#### 3.3.2 Function and modification of NBS1

NBS1 is an essential part of the MRN complex and controls the ATM and ATR‐mediated DDR pathways.^91^ To maintain genomic integrity, the MRN complex's NBS1 component coordinates DSB repair and checkpoint signaling through unrecognized interactions with ATM, MDC1, and Sae2/Ctp1/CtIP. Notably, the Ctp1 pThr‐Asp motif is bound by the NBS1 FHA domain, which recruits phosphorylated Ctp1 to DSBs.^90^ RNF20 (chromatin remodeling), RAD18 (translesion DNA synthesis, TLS), ATM, RPA (cell cycle checkpoint), and MRE11 (HR and NHEJ) are a few of the signaling pathways that are regulated by NBS1 after DSB introduction.^92^ For instance, the MRE11 nuclease is directly recruited to DSB sites by the NBS1 C‐terminus, hence MRE11 nuclease accumulation is eliminated in NBS1 clones lacking the MRE11‐binding region.^92^ During the S and G2 stages of the cell cycle, CDK‐mediated phosphorylation at S432 controls NBS1 activity. The mutation of NBS1 at the CDK phosphorylation site dramatically decreased the production of ssDNA tails in S phase and G2 phase.^92^ Additionally, NBS1 controls ATM kinase activity and serves as a signal sensor of ATM‐dependent cell cycle checkpoint activation.^93^ Specifically, NBS1 is a key regulator of ATM activation following DSB introduction, as indicated by the observation of numerous abnormalities in ATM‐related responses, such as H2AX phosphorylation, in NBS1‐deficient cells. Therefore, through alterations like H2AX phosphorylation and H2A/H2AX UB, as well as RNF20‐dependent H2B UB, NBS1 may play a significant role in the DDR.^93^, ^94^ NBS1 promotes ATR activation through its interaction with RPA32 and TopBP1. NBS1 is also critical for secondary ATR‐dependent phosphorylation, such as that of RPA, depletion of NBS1 significantly reduced RPA phosphorylation at S33 after camptothecin treatment.^93^ According to in vitro ATR kinase tests, the N‐terminal domain of NBS1 independently activates ATR without the assistance of TOPBP1.^95^

NBS1 may interact with nucleolar proteins by interacting with γ‐H2AX, thereby playing a role in clearing damaged H2A/H2B nucleosomes and subsequently promoting HR repair. NBS1 may also involve in histone modification and KAP1/CHD3‐dependent chromatin remodeling to promote efficient DSB repair.^93^ Through its FHA domain, NBS1 binds to phosphorylated CtIP (yeast Sae2 or Ctp1) for subsequent DNA end resection in HR repair.^94^ Human MR complexes have been shown to bind to DNA more readily when NBS1 is present, and they have also been demonstrated to more quickly cleave properly paired hairpin ends when ATP is present. The catalytic MR head's ATP‐bound closed state, which is essential for activating ATM kinase, is stabilized by NBS1. The human MR complex interacts with NBS1 to control the intranuclear hydrolysis of protein‐blocked ends of DSBs.^74^ Human heterochromatin protein 1 (HP1) has been found to interact with NBS1, and similar to its effect on *Drosophila*, short interfering RNA‐mediated NBS1 inhibition reduced HP1α levels in human cultured cells.^91^ Additionally, the choice of repair pathway following induced telomere disruption is determined by the phosphorylation of NBS1. NBS1 is dissociated from TRF2 by CDK2 during the S/G2 phase, which also encourages the development of the TRF2 Apollo/SNM1B complex and safeguards the leading strand telomere. The association of dephosphorylated NBAS1 S432 with TRF2 increases the replacement of deleted POT1–TPP1 telomeres following NHEJ repair, and the traditional NHEJ‐mediated repair of deleted TRF2 telomeres requires phosphorylated NBS1S432 to activate ATM.^96^

ADP ribosylation, a special PTM, is essential to several biological processes, including the DDR. The basic poly (‐ADP) ribose unit of a ribosome is recognized by the BRCT structural area of NBS1. The movement of these domain‐containing proteins to DNA damage sites and the promotion of the DDR are mediated by the interaction between poly (‐ADP) ribose and FHA or BRCT structures. Additionally, the interaction between poly (‐ADP) ribose and the NBS1 BRCT domain during DDR and ATM‐dependent cell cycle checkpoint activation is crucial for the early activation of ATM.^97^

NBS1 may also mediate NHEJ repair via the RNF20‐dependent UB of H2B, independent of 53BP1.^93^ According to research, the Skp2 E3 ligase interacts with NBS1 at DSBs to activate its K63‐linked UB, which is essential for the connection between NBS1 and ATM and facilitates ATM recruitment to DNA foci for activation.^98^ To encourage ATM activation, Skp2 ubiquitinates NBS1 at its C‐terminus (K735 and K751 sites). The successful recruitment and stable interaction of NBS1 with DSBs are dependent on the rnf8‐mediated UB of NBS1, according to an experiment based on the NBS1–K435R mutant. These two UB processes are crucial for DSB repair carried out by HR. Notably, distinct mechanisms that control NBS1's UB control its roles in DSB repair and checkpoint activation. Different regulatory processes in HR‐mediated DSB repair may be influenced by multiple levels of NBS1 UB, allowing for more accurate and efficient DSB repair in response to cell damage signals.^99^ However, except for phosphorylation and UB, little is known about other PTMs of NBS1 or their regulatory effects on NBS1. Therefore, we need to further study other NBS1 PTMs and the various biological functions of NBS1 to better understand all the important roles NBS1 plays in DNA damage repair (Figure 5B).

## 4 KU70/80 HETERODIMER IN NHEJ REPAIR

The NHEJ pathway is regulated and participated in by the Ku70/80 heterodimer, which is the central protein complex in the route, when DNA DSB damage occurs. The DNA‐dependent protein kinase catalytic subunits (DNA–PKcs), X‐ray cross‐complementary protein 4 (XRCC4), XRCC4‐like factor (XLF), and ligase IV are a few additional essential proteins that participate in the NHEJ pathway. These fundamental proteins ligate broken DNA ends and bind DSBs. But in the NHEJ repair pathway, a Ku70/80 heterodimer made of Ku70 and Ku80 is the first to detect a DSB.^100^, ^101^, ^102^, ^103^, ^104^ High attraction for DNA ends is displayed by the DNA–PKcs catalytic subunits, and this affinity increases after the Ku70/80 heterodimer binds to DSB ends.^105^ In particular, the identification of a DSB by a Ku70/80 heterodimer, which subsequently attracts a DNA–PKcs, forms an active DNA‐dependent protein kinase holoenzyme complex (DNA–PK), is the first step in NHEJ repair.^106^ When NHEJ is necessary for DNA excision, the DNA–PKcs is attracted to the end of the DNA break that is bound by a Ku70/80 heterodimer in complex with the Artemis nuclease. After autophosphorylating DNA–PKcs, Artemis is activated. Artemis then cleaves DNA ends at ssDNA‐double‐stranded DNA (ss‐dsDNA) boundaries, including all overhangs and other structures, like gaps, loops, and bubbles brought on by incompatibility between the two strands, allowing the broken DNA ends to be joined.^102^, ^105^, ^107^

### 4.1 Ku70/80 heterodimer

#### 4.1.1 Structure of the Ku70/80 heterodimer

The Ku 70/80 heterodimeric protein is a crucial regulator of DNA DSB repair in the NHEJ repair process and is the first complex to bind to DSB sites.^103^, ^108^

**FIGURE 6:**
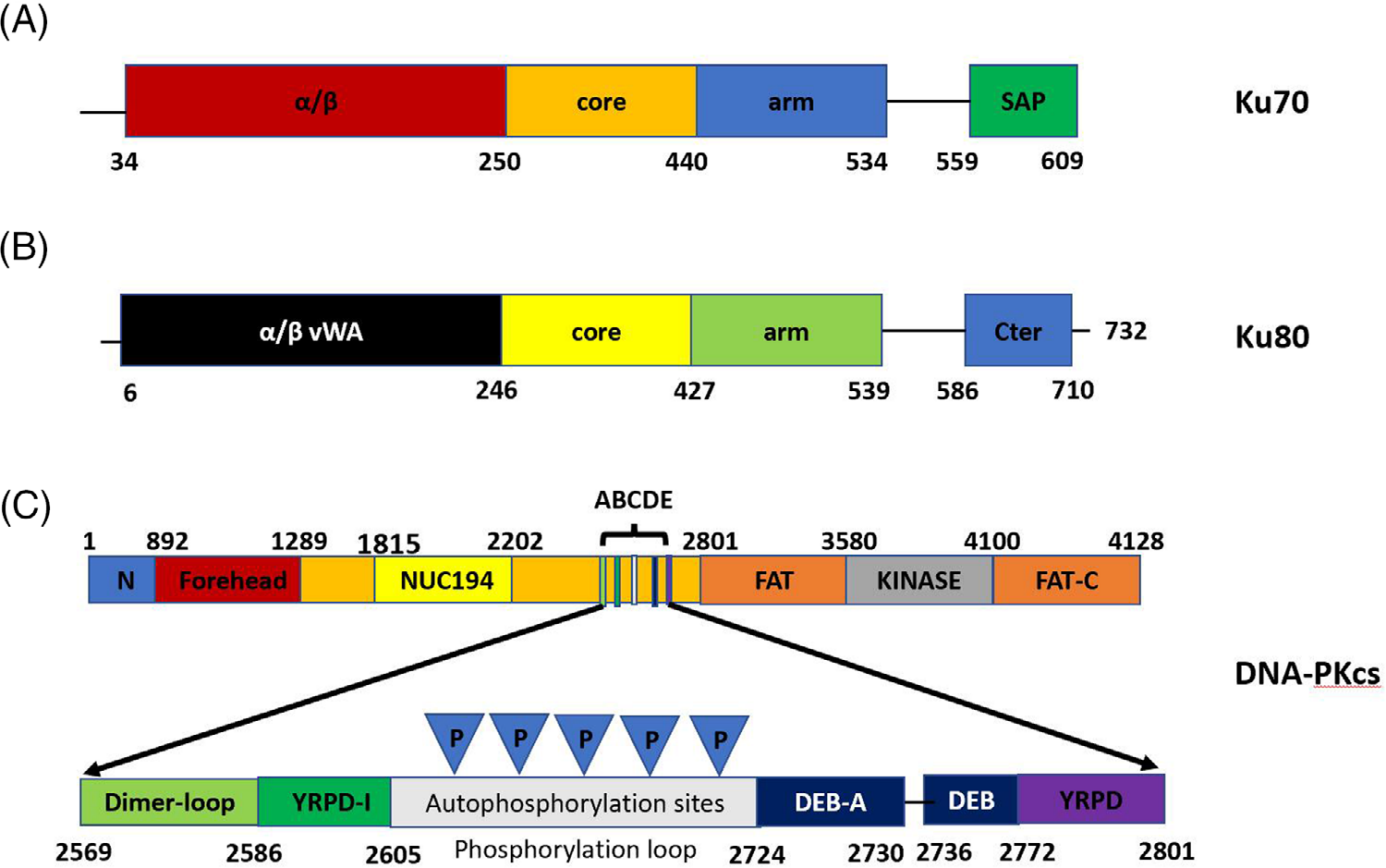
The Ku70/80 heterodimer in human beings. The C‐terminal region of the monomers has a few mutation sites. (A) Domain of Ku70. (B) Domain of Ku80. (C) Domain of DNA–PKcs.

Ku70 (69 KD) and Ku80 (83 KD) form the Ku70/80 heterodimer, which has a circular structure and a central tube suitable for ds‐DNA helix formation. The Ku70/80 heterodimer consequently has significant affinity for the ends of ds‐DNA. The Ku70/80 heterodimer immediately binds to the end of the DNA upon the introduction of a DSB, enlisting and activating DNA–PKcs to the injury site. The Ku70/80‐DNA–PKcs (DNA–PK) complex places the DNA terminal into a synaptic complex and guards it against deterioration.^104^ The N‐terminal “/” domains, core domains (bails, columns, and bridges) from the von Willebrand family, a C‐terminal arm, and a helical CTD are shared by the human Ku70 and Ku80 subunits.^7^, ^104^ The Ku loop structure is preformed and extremely stable, according to the wide interface between the Ku70 and Ku80 subunits and the similarity between the distinct crystal structures of the Ku70/80 heterodimer and Ku70/80 heterodimer–DNA complex. The mutual stability of the two Ku subunits on cellular nucleotides has been proven by a publication. In fact, it is possible that the adaptable Ku80 CTD, which extends from the DNA‐binding core of Ku and has sections from the 545 to the 732‐residue position, is crucial for keeping DNA–PKcs at a DSB.^7^ The Ku70/80 heterodimer is a circular shape with a central channel that is strongly linked to the DNA helix, as the crystal structure demonstrates. According to research, the Ku70/80 loop slips over DNA ends regardless of the nucleotide sequence pattern, which is a special property that accounts for the Ku70/80 heterodimer's strong affinity for DNA ends. Most experts agree that the Ku–DNA complex serves as the main scaffold for the activation and recruitment of all other NHEJ core proteins, including DNA–PKcs, the XRCC4–ligase IV complex, XLF, and a number of processing enzymes.^108^ Different CTRs are carried by Ku70 and Ku80 (a 7KD SAP domain in Ku70 and a 17KD center in Ku80).^109^ Different CTRs are carried by Ku70 and Ku80 (a 7KD SAP domain in Ku70 and a 17KD center in Ku80)^103^ (Figures 6A and B).

#### 4.1.2 Function and modification of Ku70/80 heterodimer

The Ku70/80 heterodimers that bind to the ends of the DNA in DSBs start the NHEJ pathway. Numerous DNA ends, such as blunt ends, hairpin‐shaped ends, and ends with protruding single‐strand overhangs, are recognized by the Ku70/80 heterodimer.^103^ When the Ku70/80 heterodimer attaches to DNA, it changes conformation to create the Ku–DNA complex. This complex then draws DNA–PKcs to the DSB site, where it binds and produces the DNA–PK holoenzyme.^110^ In the course of NHEJ repair, the Ku70/80 heterodimer creates a loop, binds to the ends of DSBs, and serves as a scaffold to draw in more NHEJ‐associated elements. The ubiquitin E3 ligase RNF8 ubiquitinates Ku80 after NHEJ repair is finished, which results in the Ku70/80 heterodimer loss from DNA in mammalian cells.^111^ In particular, a AAA+ ATPase known as Valosin‐containing protein (VCP)/p97 and its cofactors Udf1‐ and Npl4 remove the Ku70/80 heterodimer following UB.^103^

A Ku70/80 heterodimer recruits several classical (c)‐NHEJ factors including a Ku‐binding motif to DSBs.^103^ It is well known that the Ku70/80 heterodimer shields free DNA ends from exonuclease attack. The alternative end‐joining pathway's MRN complex‐dependent end resection step is significantly inhibited in cells by the Ku70/80 heterodimer.^103^ RNA hairpins and RNA–DNA hybrids are both recognized by the Ku70/80 heterodimer. Additionally, it makes it easier for DNA ends to attach to other c‐NHEJ components. The telomeric dsDNA ends are directly bound by the Ku70/80 heterodimer, which also shields the telomeres from 5′ excision. Through interactions with the telomerase TLC1 RNA component and the telomere factor Sir4, it is also involved in telomerase recruitment. Numerous PTMs that the Ku70/80 heterodimer carries may regulate how well it recognizes DNA.

The Ku subunits are phosphorylated in vitro by DNA–PKcs at Ser6 and S577 of Ku70 and at S580 and T715 of Ku80, according to research from the Lees‐Miller lab. It has been demonstrated that variations of the Ku70/80 heterodimer with alanine substitution mutations at these five sites are completely capable of granting cells complementary radiosensitivity. DNA–PK‐dependent phosphorylation of the Ku70/80 heterodimer is not required for c‐NHEJ. Another study suggested that the separation of the heterodimer from DSBs in cells was aided by the phosphorylation of the Ku70/80 heterodimer subunits.

**FIGURE 7:**
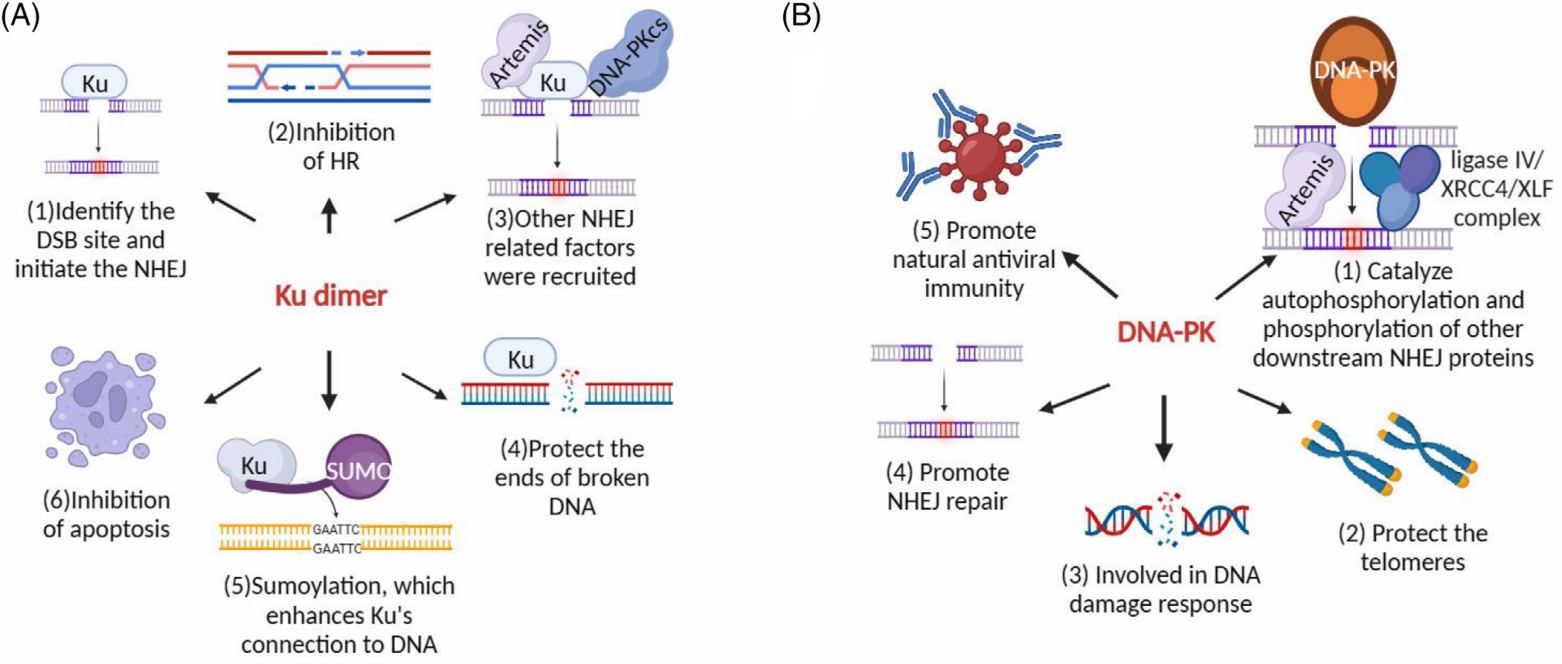
Biological function and modification of the Ku70/80 heterodimer and DNA–PK. (A) (1) The Ku70/80 heterodimer starts the nonhomologous end joining (NHEJ) repair mechanism by identifying and binding to DNA breaks. (2) The Ku70/80 heterodimer inhibits MRN‐dependent terminal DNA excision, thereby inhibiting homologous recombination (HR). (3) NHEJ‐related factors are attracted to DSBs by the Ku70/80 heterodimer. (4) Ku70/80 heterodimers protect the ends of broken DNA. (5) DNA‐binding of the Ku70/80 heterodimer is improved by SUMOylation. (6) The acetylation of the Ku70/80 heterodimer can inhibit cell apoptosis. (B) (1) In addition to phosphorylating downstream NHEJ proteins, DNA–PK also catalyzes their autophosphorylation. (2) A telomere's end is shielded from removal by DNA–PK. (3) DNA damage response (DDR) involves DNA–PK phosphorylation. (4) NHEJ repair is promoted by DNA–PK. (5) DNA–PK deletion reduces the phosphorylation rate of cGAS and promotes the natural antiviral immune response, effectively limiting viral replication and enhancing natural antiviral immunity mediated by cGAS.

In budding yeast, the CTR of Ku70 is SUMOylated, which enhances Ku70 interaction with DNA. The Ku70 and Ku80 subunits were found to be SUMOylated as a result of site‐specific mapping of human SUMO alterations. The poly (ADP‐ribosylation) of the Ku70/80 heterodimer decreased the affinity of the Ku70/80 heterodimer for DNA, according to an electrophoresis mobility shift test. Hochegger et al. observed that Parp‐1 blocked Ku70/80 heterodimer binding to DSBs in light of this finding, allowing HR repair pathway chemicals to reach damaged DNA. It has been demonstrated that acetylation of the Ku70/80 heterodimer subunits modifies its interaction with Bax, which prevents apoptosis by keeping Bax out of mitochondria.^103^ The disruption of the connection between the Ku70/80 heterodimer and the p53 mRNA by the Ku70/80 heterodimer subunit improves the Ku70/80 heterodimer‐mediated inhibition of p53 mRNA translation under genotoxic stress conditions. Reduced cysteine is required for the engagement of the Ku70/80 heterodimer with DNA, and the alkylation of N‐ethylmaleimide blocks this contact. It has been demonstrated that the endogenous metabolite inositol 6‐phosphate binds to Ku70/80 heterodimers and activates the c‐NHEJ pathway.^103^ The Ku70/80 heterodimer is involved in the regulation of ATM and ATR signaling pathways in DNA DSB repair and can control the activity of ATM and other phosphatidylinositol (PI) 3‐related kinases as well as the phosphorylation of p53 (S18) during DSB detection.^112^ Although the Ku70/80 heterodimer recognizes DSB sites, guards broken DNA ends, and takes part in related signaling cascades, more research on the heterodimer's structure and function is crucial for better understanding the DDR's mechanism (Figure 7A).

### 4.2 DNA–PK complex

#### 4.2.1 Structure of DNA–PK

At the conclusion of a DNA strand break, DNA–PKcs and the Ku70/80 heterodimer combine to generate DNA‐dependent protein kinase (DNA–PK).^113^, ^114^ The biggest serine/threonine protein kinase in the PIKK family and the most highly expressed PIKK in human cells is the DNA‐dependent protein kinase catalytic subunit DNA–PKcs, which is represented by the PRKDC gene. The Ku70/80 heterodimer in the NHEJ pathway attracts DNA–PKcs to DSB sites, effectively controlling and repairing ionizing radiation‐induced DNA DSBs.^115^ DNA–PKcs is a crucial enzyme in the DNA NHEJ process, which can result in DNA misalignment and unintended DNA breaks.^116^ DNA–PKcs homodimers are created when the core domain of DNA–PKcs interacts with the CTD of Ku80.^110^ Like all PIKKs, DNA–PKcs is made up of a solenoidal structure made up of HEAT repeats and a conserved FAT and kinase domain (FATKIN) (Figure 6C). The three DNA–PKcs domains are N‐HEAT (aa 1−872), M‐HEAT (aa 890−2580), and FATKIN (aa 2800−4128), according to recognized convention.

DNA–PK functions as a holoenzyme that detects and locates DNA ends more effectively than either component working independently. However, Ku70/80 heterodimers must first be loaded onto damaged DNA ends due to the tight topology of DNA–PK. DNA facilitates and strengthens the interaction between Ku70 and DNA–PKcs, whether the Ku70/80 heterodimer finds DNA ends alone or in combination with DNA–PKcs. This allows the folded domain of the Ku80 CTR to bind with DNA–PKcs, which activates DNA–PKcs.^116^ DNA–PKcs, Ku70, Ku80, and dsDNA combine to create a 650Kd heterotetramer in the composite structure with a stoichiometric ratio of 1:1:1:1. DNA–PKcs's N‐terminal ‐solenoid (around 2800 residues) folds into a double loop, which connects to both the Ku70/80‐DNA complex and the catalytic core domain. Together, DNA–PKcs and Ku70/80 heterodimers create a DNA‐binding tunnel that covers 30 bp of DNA. Because this tunnel inhibits DNA–PKcs and dsDNA from sliding inwards toward one another, it appears to shield the ends of fragmented DNA from needless processing. According to structural and biochemical investigations, DNA–PKcs undergoes conformational changes and has its kinase activity allosterically stimulated by the Ku70/80 heterodimer. We put forth a concept for how DNA–PKcs is activated in which allosteric signals are produced during the holoenzyme assembly of DNA–PK and are then transmitted to the kinase domain via the N‐terminal HEAT repeat sequence and FAT domain. We hypothesized a mechanism for the recognition and protection of damaged DNA ends based on our study, offering a structural basis for comprehension of DNA–PKcs and DNA–PK‐mediated activation of the NHEJ pathway.^117^

#### 4.2.2 Function and modification of DNA–PKcs

Activated by binding to damaged DNA ends, DNA–PKcs is a serine/threonine protein kinase that catalyzes the autophosphorylation and phosphorylation of other downstream NHEJ proteins, including processing proteins like the nuclease Artemis. The ligase IV/XRCC4/XLF complex is attracted to the damaged DNA ends and catalyzes the ligation of DSBs following DNA–PK processing of DNA ends.^110^ DNA–PKcs participates in mammalian telomere protection, transcription and other cellular processes, including some processes in which dependence on its kinase activity remains unclear.^118^ When the DNA–PK complex is assembled at a DSB, two DNA–PKcs molecules are trans‐autophosphorylated across the DSB, which breaks up the DNA–PK complex in vitro. Numerous sites have been discovered as DNA–PKcs autophosphorylation sites, including S2056, the ABCDE or T2609 cluster, which is a collection of sites between residues 2609 and 2647, and T3950. The ABCDE site exhibits protein kinase activity, but after the occurrence of alanine substitution mutations, DNA–PKcs's ability to dissociate from the Ku–DNA complex is diminished, suggesting that phosphorylation at the ABCDE site is involved in controlling the disassembly of the initial DNA–PK complex and plays a significant role in DSB repair.^106^ Multiple sites, including S2056, the ABCDE cluster, and T3950, are phosphorylated in vivo, prompting DNA–PKcs to respond to DNA damage. Because cells expressing DNA–PKcs are unable to autophosphorylate at the ABCDE/T2609 cluster, they are extremely radiosensitive and have several DSB repair errors. Furthermore, mutant DNA–PKcs carrying an autophosphorylation‐deficient ABCDE cluster or mutations involving the replacement of S2056 with an alanine residue or rendering DNA–PKcs kinase dead are retained longer at DNA damage sites in vivo than wild‐type DNA–PKcs.^106^ The area between aa 2581 and 2783 contains the ABCDE cluster's well‐studied DNA–PKcs autophosphorylation hotspot (aa 2609−2647). ABCDE clusters may be cis‐phosphorylated and encourage subsequent NHEJ pathway events close to the DNA–PKcs kinase activity site in association with DNA.

The most unusual of these phosphorylation clusters is PQR, which spans the aa residues 2023−2056. PQR is the only DNA–PKcs HEAT repeat that lacks a sequential folding pattern among the rest.^116^ The functions related to the other phosphorylation sites (except S3205) within the ordered region of DNA–PKcs can be explained. The S72 site is close to the Ku bridge, and its phosphorylation prevents DNA–PKcs from interacting with the Ku70/80 heterodimer, which in turn prevents DNA–PK from binding to DNA. The kinase activity of DNA–PKcs has been reported to be inactivated by phosphorylation at S72. The cumulative effect of nearby ABCDE clusters in repair partner selection may be responsible for the phosphorylation at T946 and S1003, which has no effect on DNA–PKcs kinase activity but suppresses NHEJ pathway activation. The T3950 site is buried in the accessible structure of the kinase's activation loop and undergoes a conformational shift before it can be phosphorylated to control DNA–PK's action.^116^ A lack of DNA–PK efficiently restricts viral replication, boosts the natural antiviral immune response, and enhances natural antiviral immunity mediated by cGAS, according to a study.^119^ In conclusion, DNA–PK is a major player in numerous physiological pathways, the repair of DNA DSBs, and the maintenance of genome integrity. Additionally, it has been demonstrated that DNA–PK controls transcription, takes part in the maturation of the immune system, and shields telomeres.^120^ The investigation of DNA–PK's undiscovered functions will help us better comprehend the role of DNA–PK in the process of DNA damage repair based on what is known about the structure and function of this protein (Figure 7B).

## 5 SELECTION OF THE HR OR NHEJ PATHWAY VIA COMPETITIVE COORDINATION OF THE MRN COMPLEX AND THE KU70/80 HETERODIMER

### 5.1 HR or NHEJ repair pathway choice

The two primary repair mechanisms for DSB damage, HR and NHEJ, either compete or work together. The functions played by NHEJ and HR in the repair of DSBs are complimentary. In G2 phase, NHEJ is assumed to be the preferred approach and can rapidly repair approximately 80% of X‐ray‐induced DSBs, while other DSBs are terminally removed and repaired by HR. It is believed that the repair of DSBs via NHEJ is first attempted in G2, but if reconnection in delayed, HR resection and repair will occur.^121^ Competitive and complementary relationships between the NHEJ and HR pathways at DSB sites have been identified in rice. The inhibition of the NHEJ pathway may increase the rate of HR‐related sequence‐specific integration, while NHEJ inhibits random integration, resulting in a synergistic effect.^122^

NHEJ is active in the G1, S, and G2 phases of the cell cycle, but it is most crucial in the G1 phase just before chromatid replication, while HR predominates late in the S/G2 phase. However, the possibility that these two routes will compete in the DDR is increased by their overlap.^123^, ^124^ There is competition between HR and NHEJ; however, the nature of this competition has been ambiguous. HR and NHEJ are likely to engage in passive competition, depending on whether the HR or NHEJ protein first combines with DSB sites or homologous sister chromatids. If competition is passive and neither repair pathway is saturated, elimination of one pathway shifts DSB repair to the other. In addition, HR and NHEJ may be in interactive competition; that is, they may interact with each other and affect each other's activity. For instance, DNA–PKcs not only participates in NHEJ but also phosphorylates protein substrates like ATM and WRN that participate in HR. MRN complexes can also affect both HR and NHEJ.^125^ Similar to how early players battle to bind DSBs, investigations have shown that HR and NHEJ are both active in the S and G2 stages of the cell cycle. The Ku70/80 heterodimer is recruited to DSBs more quickly after the induction of DSBs than HR‐related repair factors, allowing NHEJ to disrupt HR. This work also shown that the binding of the Ku protein (rather than other NHEJ components) to DSBs may slightly interfere with the initiation of HR, leading to competition between HR and NHEJ in DSBs caused by ionizing radiation or endonuclease I‐SceI. A DSB repair bias in favor of HR is shown in cells lacking NHEJ genes, indicating that both repair mechanisms are capable of mending the same break.^124^ NHEJ and HR are two main repair pathways that ensure genomic stability in the chromatin environment.^126^ A cell's reliance on error‐prone DNA repair mechanisms, such as NHEJ for DSB repair, is impacted by any disruption to HR, which raises the risk of genomic instability.^127^ NHEJ is promoted by p53‐binding protein 1 (53BP1) by promoting the connection of distal broken DNA ends, while HR is antagonized by 53BP1, which inhibits DNA end excision.^128^ BRCA1 and 53BP1 antagonize each other in DSB repair pathway choice. 53BP1 can form a barrier that inhibits DSB end resection, thereby inhibiting HR and promoting NHEJ. In contrast, BRCA1 promotes HR by removing the barrier to 53BP1 formation, thereby activating DSB end resection.^129^, ^130^, ^131^, ^132^

### 5.2 Competitive coordination of the MRN complex and the Ku70/80 heterodimer

The main complexes in the NHEJ and HR repair pathways, the MRN complex and the Ku70/80 heterodimer, respectively, play significant roles in the beginning and control of the repair pathways. The Ku70/80 heterodimer joins with a DSB end after the introduction of a DSB to prevent the DNA end from being degraded, initiating the NHEJ pathway and bringing in additional NHEJ components, like DNA ligase IV, to repair. One of the primary protein complexes in the HR repair pathway and one of the protein complexes essential for the recognition, associated signaling, and repair of DNA DSBs is the evolutionarily conserved MRE11–RAD50–NBS1/XRS2 complex (*S. cerevisiae*). The MRX (*S. cerevisiae*)/MRN complex was quickly attracted to a DSB and began DSB resection while maintaining DSB ends linked for repair. To coordinate DSB repair and cell cycle events, the MRX (*S. cerevisiae*)/MRN complex also requires the recruitment and activation of protein kinase Tel1 (ATM in mammals).^42^

During DSB repair, the Ku70/80 heterodimer competitively interferes with the HR repair pathway. Research results have shown that the relative radioresistance caused by Ku70 deletion is due to the HR‐dependent repair pathway. The Ku70/80 heterodimer can interfere with HR‐mediated DSB repair and may compete with HR for DSB recognition. However, the Ku protein does not inhibit all HR reactions but specifically interferes with HR‐mediated DSB repair.^123^ The HR‐related genes OsRAD51A2 and BRCA1 are impacted, indicating that OsKu70 may also have a role in regulating the HR repair pathway.^122^ The information also revealed that the Ku70/80 heterodimer binds to DSBs during all phases of the cell cycle and may actively be translocated from DSB ends, allowing for DNA end resection and HR. Additionally, when DNA ends are occluded by Mt‐Ku, the subsequent recruitment of HR factors to DSBs and DNA end resection are decreased. Finally, the data indicated that during HR‐mediated DSB repair, significantly fewer cells exhibit persistent DSB binding to Mt‐Ku (14.45%) than Ku70/80 heterodimer‐deficient cells (32.05%).^133^ These findings indicate that the Ku70/80 heterodimer has a high affinity for DNA ends and may attach to DSB ends fast, shielding them from processing and possibly inhibiting HR by blocking the assembly of HR‐related repair components at the damage site.^124^

The HR repair pathway is chosen because Ku70/80 heterodimers and MRN complexes compete to block the NHEJ repair pathway, while Ku70/80 heterodimers and MRN complexes preferentially block the HR repair pathway. When telomeres are defective in the late stages of the G2 phase, it has been demonstrated that the MRN complex can impede the NHEJ pathway.^134^ Each MRN complex component, along with the loss of the Ku70/80 heterodimer and DNA ligase 4, inhibits NHEJ repair to the same degree. The MRN complex's primary function is to bring separated DNA ends together, and the MRE11 dimerization domain must be intact for DNA damage repair.^135^ These findings have persuasively demonstrated that MRN complexes can play important regulatory roles in the NHEJ repair pathway. These activities can be separated by identifying MRE11 nuclease‐deficient alleles, which are related but distinct functionalities that MRN complexes can exhibit in various NHEJ processes.^135^ The MRN complex variation is required for the NHEJ repair system in *Streptococcus pombe* to cleave DNA ends. The repair of DBSs in *Streptococcus pombe* is performed by the NHEJ pathway and requires the MRN complex. The results of genetic analyses have indicated that the MRN complex recruits an unknown hairpin‐opening nuclease during an abnormal NHEJ response.^136^ The MRN complex connects the loose ends of DNA to enable efficient NHEJ repair. It has been demonstrated that the MRN complex repairs the NHEJ process in fission yeast by binding damaged telomeres. NHEJ activity at telomeres was blocked by disruption of the MRN (RAD23 (MRE11)–RAD50–NBS1) complex in *S. cerevisiae*.^137^ Etoposide‐induced DSBs are repaired via the NHEJ pathway in human cells where the cell cycle is halted in the G0/G1 phase. Surprisingly, this process requires the involvement of the MRN complex (a direct homologue of MRX) and CtIP.^138^

In the DNA DSB repair process, there is collaboration as well as rivalry between the MRN complex and the Ku70/80 heterodimer. The MRN complex may be able to attach to DNA ends that the Ku70/80 heterodimer has previously engaged, generating a component loop that is unique to DNA ends and unsuitable for internal DNA binding, according to data acquired from mammalian cell tests. The Ku70/80 heterodimer reduces the need for the MRN complex to “clean” DNA ends in budding yeast to prepare for DNA end resection, indicating that the Ku and MRN complexes compete for binding to DSB ends.^42^ Experimental evidence suggests that the MRE11 complex overcomes the Ku70/80 heterodimer‐induced barrier through a nickel‐dependent excision mechanism. Together with Sae2/CtIP, the MRE11 complex excises a brief stretch that is essential for removing the Ku70/80 heterodimer from the terminal DNA, resulting in a more intricate and useful connection between the NHEJ and HR pathways. This mechanism is evident in human cells at single‐ended DSBs generated at broken DNA replication forks.^139^ Studies conducted in vitro have revealed that the MRN complex controls the dissociation of the Ku70/80 heterodimer from DNA ends.^133^

There is ongoing discussion about whether the MRN complex and the Ku70/80 heterodimer bind to the same DSB site, the relative order of this binding, and whether this competition endures across the whole DDR to DSBs. Computer modeling supports the so‐called “wound path,” which involves multipoint crosstalk between the Ku70/80 heterodimer and MRN complex during loading, not competition or sequential attachment.^140^ The Ku70/80 heterodimer must be dissociated during the delayed response of HR, and Ku70/80 heterodimer dissociation is achieved via a combination of nucleolytic and proteolytic functions. Interestingly, the major nuclease involved in Ku70/80 heterodimer dissociation is MRE11, suggesting crosstalk between these two DNA end‐binding complexes.^140^ In other words, in the absence of Ku, MRE11 binding to DNA ends rises in cells in the G1 phase and the number of ends that are excised increases. It has been demonstrated that the MRN complex and the Ku70/80 heterodimer compete for binding to DNA ends. In contrast, excessive Ku70/80 heterodimers inhibit the binding of MRE11 to DSBs and the initiation of DNA resection in G2‐arrested cells.^141^ Biochemical data have suggested that the MRN complex prevents Ku70/80 heterodimers from being recruited to DSB sites, and when a Ku70/80 heterodimer binds to a DSB, the MRN complex actively displaces it, which is followed by DNA end resection.^133^

Both in vivo and in extracts, the MRN/MRX (*S. cerevisiae*) complex is connected to DSB end resection. Studies of pure proteins conducted in vitro have demonstrated that the MRN/MRX (*S. cerevisiae*) complex enhances the release of Ku70/80 heterodimer inhibition by recruiting distant enzymes both inside and outside the nucleus. The same study also discovered that the MRN complex stimulates high production of Exo1 from DNA in a dose‐dependent manner when the Ku70/80 heterodimer and DNA–PKcs are present. By enlisting Exo1 and increasing DNA–PKcs autophosphorylation, the MRN complex stimulates resection in the presence of the Ku70/80 heterodimer and DNA–PKcs, but it prevents the terminal reconnection induced by these two molecules.^142^

### 5.3 Competitive coordination of ATM and DNA–PK

The interaction between the downstream signaling molecules DNA–PK and ATM reveals both the competition and cooperation between the MRN complex and the Ku70/80 heterodimer. For instance, DNA–PKcs can be phosphorylated by ATM at T4102, which stabilizes the association between the Ku–DNA complex and NHEJ repair mechanisms at DNA DSBs.^143^ Additionally, in single‐ended DSBs, ATM activity is necessary for the NHEJ repair pathway to release Ku70/80 heterodimers and DNA–PKcs from DSBs. Thus, aberrant interactions between single‐ended DSBs and Lig4‐dependent end joining that subsequently accompany DNA–PKcs autophosphorylation are prevented.^144^ Along with ATR and ATM, DNA–PK, a marker protein for NHEJ, was discovered to serve a regulatory role in CPT‐induced DSB repair. This regulation is achieved by the cooperative phosphorylation of p53 and RPA. Following DNA damage, DNA–PK concurrently phosphorylates RPA at the N‐terminus of the RPA32 subunit, and in a Chk1/Chk2‐independent manner, ATR and ATM phosphorylate Ser37 and Ser46 of p53, respectively. This releases p53 and RPA from the p53–RPA complex, which controls the HR repair pathway. Therefore, our result indicates a unique crosstalk mechanism between the HR and NHEJ repair pathways as well as cooperation between DNA–PK and the ATM/ATR/p53 checkpoints.^145^

In many different cell types, DNA–PKcs regulates the amount of ATM. Additionally, DNA–PKcs and ATM both phosphorylate the DNA–PKcs T2609 cluster, which is crucial for NHEJ and HR, indicating that both proteins are also involved in DSB repair. The fact that DNA–PKcs can regulate ATM levels without DNA–PKcs kinase activity raises the possibility that ATM stabilization is not always dependent on DNA–PKcs‐mediated phosphorylation of ATM or other targets. The phosphorylation of DNA–PKcs by ATM and the regulation of ATM levels by DNA–PKcs indicate that these proteins cooperate to control the efficiency and choice of DSB repair pathways, and that ATM facilitates HR‐mediated repair of DSBs by phosphorylating DNA–PKcs and HR factors that are unrelated to replication.^146^ The downstream signaling molecules ATM and DNA–PK continue to compete with and coordinate with the MRN complex and the Ku70/80 heterodimer, controlling the selection of DSB repair pathways accordingly.

The two main DSB repair pathways, HR and NHEJ, also exhibit a very complex connection as a result of the competition and cooperation between the MRN complex and the Ku70/80 heterodimer as well as their downstream signaling molecules ATM and DNA–PK.

**FIGURE 8:**
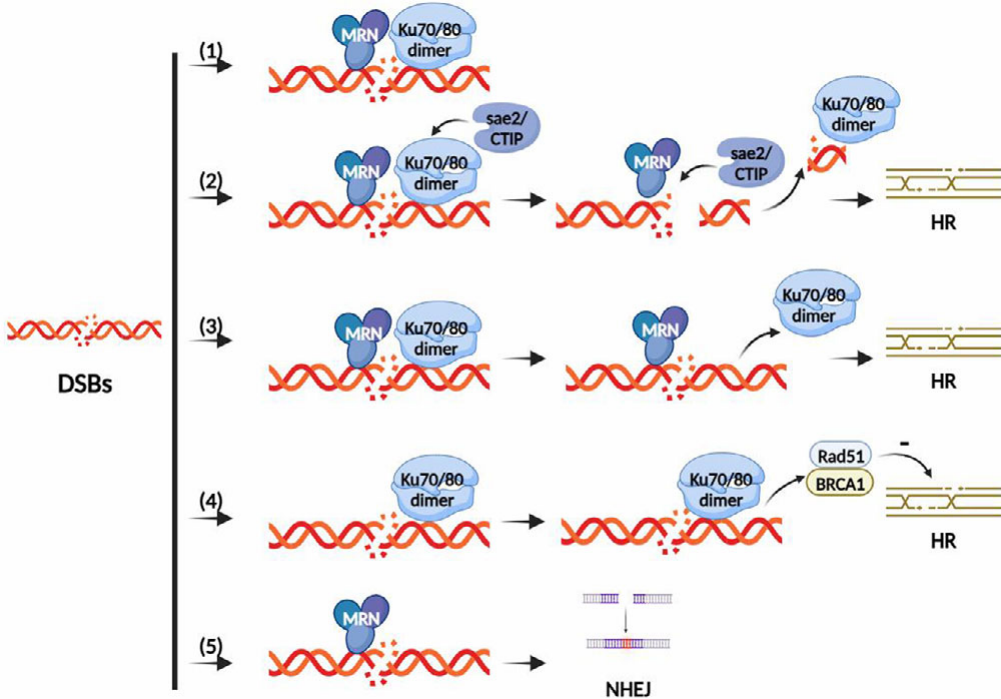
Diagram showing the competitive coordination pattern between the MRN complex and the Ku70/80 heterodimer. (1) At the end of a damaged DNA double strand, the MRN complex and the Ku70/80 heterodimer can coexist. (2) The Ku70/80 heterodimer is bound to a DNA segment by the MRN complex, which helps the MRN complex cut the end of the broken DNA double strand and promote the homologous recombination (HR) repair pathway by recruiting enzymes like Sae2/CtIP at the DSB site to cut the DNA segment. This releases the Ku70/80 heterodimer from the end of the broken DNA double strand. (3) The Ku70/80 heterodimer is actively released from the broken DNA end when the MRN complex and the Ku70/80 heterodimer unite there, allowing the MRN complex to remove the broken DNA double strand end and so facilitate the HR repair process. (4) The RAD51, BRCA1, and other proteins associated with the HR repair pathway are impacted by the Ku70/80 heterodimer, which is attached to the end of the broken DNA double strand. (5) The MRN complex can also encourage nonhomologous end joining (NHEJ) repair pathways, including DSBs caused by etoposide and dysfunctional telomere repair.

The mechanism by which the two DSB repair routes, HR and NHEJ, are chosen under particular conditions will become clearer with further research into the competing and coordinated connections between the MRN complex and the Ku70/80 heterodimer. (Figure 8).

## 6 THERAPEUTIC TARGETS FOR CANCER THERAPY

Unrepaired DNA DSBs, which are the most harmful type of cellular damage and which are highly detrimental, are sufficient to cause cell death.^147^ Cancer cells have a larger DSB load than healthy cells as a result of oncogene‐induced replication stress and flawed DDR mechanisms. Therefore, cancer cells must rely on effective DSB repair to proliferate excessively. In addition to this, radiotherapy and chemotherapy resistance is mainly due to enhanced DSB repair and ultimately leads to cancer recurrence.^148^, ^149^ Regulatory regulators have approved PAPRi as a monotherapy for BRCA1/2 mutant breast and ovarian tumors, and concepts for DNA repair‐targeted therapy in malignancies with mutations in the BRCA1 or BRCA2 tumor suppressor genes are now available.^150^, ^151^, ^152^ The two primary DSB repair mechanisms are HR and NHEJ, and by focusing on and controlling these two pathways, we may control the cellular DDR response and DSB repair, so attaining the goal of tailored therapy for cancer.^127^ It is well known that the most harmful type of DNA damage—IR‐induced DSBs—can result in chromosomal rearrangements and cell death. Therefore, to preserve the integrity of the DNA, cells have evolved a quick and efficient DDR. In addition to being able to detect DNA damage, DNA damage sensors are sensitive proteins that can also attract transducer proteins that alert enzymes to breaks in the DNA. H2AX, 53BP1, NBS1, BRCA1/2, and Ku70/80 heterodimers are just a few of the DNA damage sensor proteins that have been thoroughly described in research so far. The majority of these DNA damage sensors have the following characteristics: first, they locate at the site of DSBs within seconds or minutes of IR exposure, forming microscopically visible nuclear structural domains called IRIFs; second, the sensor proteins can modify nearby damage sites, such as by phosphorylating H2AX; third, the sensor proteins can attract other proteins to the damage sites to form protein complexes, such as MRN complexes^153^; and fourth, these DNA damage sensors can detect DNA. For instance, radiation stimulates the expression of MDC1, and MDC1 overexpression can cause DNA damage repair to activate NBS1 activity.^154^ Upstream or downstream proteins can also regulate these sensors. In the case of the human JMJD1C demethylase, interaction with RNF8 and recruitment of RAP80–BRCA1 stabilize the enzyme. Through JMJD1C binding to RNF8 and MDC1 demethylation at the Lys45 location, MDC1 increases the sensitivity of cancer cells to IR.^155^ According to one study, higher DNA breaks were linked to increased H2AX/53BP1 foci after irradiation, and there was a clear correlation between MDC1, H2AX, and 53BP1 after irradiation.^156^ Identification of these sensors following the occurrence of DSBs under IR irradiation may therefore serve as a prognostic biomarker for the effectiveness of radiotherapy in cancer patients^157^ (Table 1).

**TABLE 1:** Target proteins and corresponding inhibitors in DSB repair.

Target proteins	Inhibitors	Mechanisms	References
MRN complex	Mirin	The G2/M checkpoint is eliminated, and HR repair is compromised, when MRN complex‐dependent ATM activation and mre11‐associated exonuclease activities are inhibited.	^158^, ^159^, ^160^, ^161^, ^162^
ATM/ATR	KU‐55933	By preventing the phosphorylation of H2AX, NBS1, and Chk1, specifically inhibits ATM and makes cells more sensitive to ionizing radiation.	^158^, ^163^, ^164^
	KU‐60019	Stimulates autophagosome and p62 accumulation and inhibits autophagosome formation under autophagy‐inducing conditions; ATM inhibitors cause excessive autophagosome accumulation and cell death.	^164^, ^165^, ^166^, ^167^
	CGK733	Selectively inhibits ATM and ATR, resulting in blockade of the checkpoint signaling pathway.	^158^, ^168^, ^169^
	CP466722	Transient (4 h or less) inhibition of ATM expression was sufficient to increase the radiosensitivity of tumor cells. The inhibition of ATM and its downstream signaling pathways was equivalent to that of KU‐55933.	^158^, ^165^, ^170^, ^171^
	AZD1390	Strong selective inhibition of ATM kinase, with the level of ATM phosphorylation starting to decrease after 4 h of action; AZD1390 combined with radiation exposure resulted in G2 cell cycle block in TP53 mutant cells.	^165^, ^172^, ^173^
DNA–PKcs	NU7441	By interfering with cancer cells' DNA repair and cell cycle checkpoints, it slows down their growth and increases their radiosensitivity.	^174^, ^175^, ^176^, ^177^, ^178^
	AZD7648	AZD7648 inhibits DNA–PK, which delays DNA repair.	^174^, ^179^, ^180^, ^181^, ^182^
	M3814	M3814 encourages G2/M cell cycle arrest and death while inhibiting DNA–PKcs.	^174^, ^183^, ^184^, ^185^, ^186^
Lig 4	Rabeprazole	Specifically blocks the adenylate transfer step and DNA recombination.	^187^
	U73122	Hinders DNA recombination and the adenylate transfer process specifically.	^187^
	SCR7	SCR7 inhibits Lig4 activity and prevents DSB end joining, thereby inhibiting NHEJ repair and promoting apoptosis.	^188^, ^189^, ^190^, ^191^
PARP1	Olaparib	The removal of nucleosomes from the site of DNA damage is inhibited by blocking the recruitment of ALC1, which in turn inhibits the recruitment of RPA2 and RAD51, which can eventually hinder HR repair.	^192^, ^193^, ^194^, ^195^
	Niraparib	Promotes the accumulation of DSBs, cell cycle blockade and apoptotic cell death and inhibits the DNA damage response.	^196^, ^197^, ^198^
	Pamiparib	Selective inhibition of PARP1 with antiproliferative activity against HR‐deficient tumor cells.	^199^, ^200^, ^201^, ^202^
	Mortaparib	Inhibition and inactivation of PARP1 induces growth arrest/apoptosis signaling and tumor suppression.	^203^, ^204^
HIF‐1	Benzopyranyl 1,2,3‐triazole	Enhanced HIF‐1 hydroxylation, followed by ubiquitination and proteasomal degradation, to inhibit HIF‐1.	^205^, ^206^
	Acriflavine	It can inhibit HIF‐1 dimerization and tumor growth.	^207^, ^208^, ^209^
	Saikosaponin‐d	Inhibition of HIF‐1 increases the radiosensitivity of hepatocellular carcinoma cells under hypoxic conditions.	^210^
HDACs	Trichostatin A (TSA)	By stopping the cell cycle at the G1/G2 phase, HDAC inhibition prevents DNA damage repair.	^211^, ^212^, ^213^, ^214^
	Suberoylanilide hydroxamic acid (SAHA)	Inhibits HDACs by binding to and blocking enzyme activation sites.	^212^, ^215^, ^216^
	ITF2357	When HDACs are inhibited, the G1/S cell cycle is arrested.	^217^, ^218^, ^219^
	Panobinostat	Causes apoptosis and the arrest of the G2/M cell cycle.	^220^, ^221^, ^222^, ^223^
CDK1	AZD5438	Inhibits Cdk1 and prolongs G2/M cell cycle block.	^224^, ^225^, ^226^
Wee1	Adavosertib	Selective inhibition of Wee1 blocks DNA damage repair at the G2/M checkpoint in p53‐deficient tumors, leading to tumor cell death.	^227^, ^228^, ^229^, ^230^, ^231^
	MK‐1775	Phosphorylation of Tyr15 residues to stop cell cycle protein‐dependent kinase 1 (CDC2) activity.	^232^, ^233^, ^234^, ^235^
	PD0166285	The removal of the G2/M phase DNA damage checkpoint allows cells to enter early cell division and skip DNA damage repair.	^236^, ^237^, ^238^
CHK1	CCT244747	Cell cycle G2 checkpoint abolished.	^239^, ^240^, ^241^
	MK8776	Autophagy inhibition.	^242^, ^243^, ^244^
	LY2603618	Competitive inhibition of CHK1 with ATP results in G2/M cell cycle block and antitumor effects.	^244^, ^245^, ^246^, ^247^
CHK2	AZD7762	Promotes accumulation of DNA DSB damage, cell cycle G2/M arrest and apoptosis leading to radiosensitization.	^248^, ^249^, ^250^, ^251^, ^252^
	Honokiol	Increases apoptosis, cell cycle G2/M arrest, and DNA DSB damage buildup, all of which lead to radiosensitization.	^248^
	Tunicamycin	Encourages cell cycle G2/M arrest, apoptosis, and buildup of DNA DSB damage, which results in radiosensitization.	^248^
	CCT241533	Stops CHK2 from activating in response to DNA damage.	^253^
	VRX0466617	Blocks the recombinant Chk2 enzyme's activity, which prevents CHK2 from being phosphorylated and prevents it from being activated.	^254^, ^255^

### 6.1 Therapeutic targets in the HR pathway

Human cancers frequently have defective HR repair pathways, particularly ovarian, breast, prostate, and pancreatic cancers. These HRD cancers are susceptible to treatments that cause double‐stranded DNA breaks or replication fork arrests, such as oral small‐molecule PARPi.^256^, ^257^ HRR deficiency (HRD) is the first phenotypically defined predictive marker for PARPi therapy in ovarian cancer. BRCA1 and BRCA2 are necessary for the HR repair pathway's high‐fidelity repair of DNA DSBs. As a result, novel treatment options for high‐grade ovarian cancer are now available as a result of the discovery that PARPi disrupt the DNA repair mechanism in BRCA mutant cells. Olaparib, the first PARPi, has recently been authorized for use in ovarian cancer with BRCA mutations. Although the mutated BRCA gene alone is most associated with HRD, other essential HR proteins may also be mutated or functionally defective, potentially expanding the therapeutic scope of PARPi.^258^, ^259^, ^260^ Important proteins included in the HR repair pathway are RPA, PALB2, BRCA1, BRCA2, RAD51, MRE11, RAD50, and NBS1.

#### 6.1.1 Targeting MRN complex

The MRN complex, which was first identified in 1998 and is made up of the proteins MRE11, RAD50, and NBS1, controls the HR repair process and serves as a cell cycle checkpoint.^157^, ^261^ The HR repair pathway is launched by the MRN complex, which can also be the first to recognize DSBs and bind to them. Activated ATM and several other repair proteins are then recruited to the DNA damage site.^157^ The MRN complex is a potential target for improving the sensitivity of cancer cells to treatment because of its function in the DSB response and necessity for cell survival.^262^ Recombinant adenoviruses have been engineered to disrupt the function of NBS1, thereby increasing the sensitivity of head and neck tumors to radiotherapy.^263^ Similar to how RAD50 function disruption has been demonstrated to improve chemosensitivity in head and neck cancer.^264^ Numerous cancer types have been thoroughly researched to determine the important function of the MRN complex in DSB repair and its potential as a therapeutic target for cancer treatment: Patients with rectal, prostate, gastric, and non‐small cell lung cancer also have worse disease‐free and overall survival when they have high expression of the MRN complex, which has been linked to chemo‐ and radioresistance in breast cancer, glioma, and NSCLC.^265^, ^266^, ^267^, ^268^ Regarding their dual impact on carcinogenesis and prognosis, the effects of faulty and/or changed MRN complex expression levels are still debatable. Several DSB repair processes are modulated by MRE11 ecto‐ or endonuclease inhibitors, according to related mechanistic investigations. Cells enter the NHEJ repair pathway via HR when MRE11 endonuclease activity is inhibited, whereas repair errors occur when MRE11 exonuclease activity is blocked. These data indicate that the MRN complex is a viable target for cancer therapy; however, more work is required to create inhibitors with HR‐specific and all‐encompassing action.^269^

#### 6.1.2 Targeting BRCA1/2

In clinical settings, BRCA1 and BRCA2 are linked to hereditary breast and ovarian malignancies.^270^ Patients with prostate cancer who did not respond to prostate‐specific membrane antigen‐targeted alpha‐radiation therapy had their BRCA1 and BRCA2 genes removed, and multiple BRCA1 variations had been found.^271^ The zinc‐binding finger structural domain RING and two phosphopeptide‐binding structural domains BRCT are among the many structural domains that make up BRCA1.^272^, ^273^ Similarly, BRCA2 has multiple structural domains. The DNA binding structural domain is situated close to the C‐terminal area, while the transcriptional activation structural domain is found at the N‐terminal end. Three oligonucleotide binding folds, a tower structural domain, and a conserved helical structural domain are additional sections.^274^ BRCA1 and BRCA2 are essential for the HR pathway's ability to repair DSBs.^275^ Following radiation exposure, the BRCA1–RAP80–Abraxas complex attaches to ubiquitinated histones in response to DNA damage.^276^, ^277^, ^278^ According to a recent study, the SWI2 family members MRN and CSB can join forces with BRCA1 to create a complex in the late S/G2 phase. DNA end resection mediated by MRN is a result of interactions between BRCA1, CSB, and MRN.^279^ Furthermore, BRCA1–PALB2 interaction determines HR/single strand annealing choice and correlates with RR.^280^ These significant functions of BRCA1/2 imply that they are desirable, valuable, and sensitive diagnostic indicators for anticipating the result of radiation.^281^

#### 6.1.3 Targeting ATM/ATR

In 1967, when it was revealed that a patient with a rare autosomal recessive A‐T illness was immunocompromised, ATM was discovered through clinical case observations.^282^ Additionally, it has already been demonstrated that A‐T patients respond to radiation differently than A‐T‐negative patients.^283^ After IR exposure, A‐T cells were not only blocked in G1/S, but also could not be activated by G2/M.^284^ ATR was first identified in budding yeast, which has S and G2 checkpoint defects.^285^ ATR was found to functionally enhance the radiosensitivity of *S. cerevisiae* esr1‐1.^286^ Phosphorylation of serine or threonine residues, which have similar activities and have common substrates with ATM and ATR, is one of their primary tasks.^287^ The MRN complex, which serves as a sensor for DNA damage signaling, activates and recruits ATM to the DSB site, whereas ATR is activated and recruited to DSB sites by its stable binding partner ATRIP.^288^ The main function of ATM is to initiate a cascade of DSB signaling responses that allow the cell to phosphorylate hundreds of substrates during the DDR.^289^ For instance, when ATM activates CHK2, several sites are phosphorylated, causing apoptosis and cell cycle arrest. Cellular IR sensitivity is raised when ATM‐mediated signaling cascades are absent.^290^ It has been shown that peripheral blood lymphocytes in the quiescent state are more sensitive to radiotherapy than their normal state when ATM is inhibited.^291^ In addition, different IR dosages have been discovered to have varying effects on the synergistic connection between ATM, ATR, and DNA–PKcs. At low concentrations, ATM and ATR closely control the G2 checkpoint's interaction with CHK1, a different cell cycle mediator. The ATM–ATR complex loosens up after receiving large radiation doses, and both can independently influence the G2 checkpoint, resulting in DSB end‐resection.^292^ ATM and ATR, which are essential components of DDR and have the power to mediate cell cycle arrest and promote DDR, are thought to have the potential to dramatically increase radiotherapy's effectiveness.^293^ Preclinical research and a wealth of literature have revealed several ATM and ATR inhibitors that may radiosensitize cancer cells, but it is important to keep in mind that these agents may also be more toxic to healthy tissues. More research is needed to determine these agents' precise function in radiotherapy.

### 6.2 Therapeutic targets in the NHEJ pathway

The Ku70/80 heterodimer first identifies and binds to DNA break ends during NHEJ, and its ring structure prevents the degradation of DNA break ends and draws in other proteins like DNA–PKcs. To create the DNA–PK complex, DNA–PKcs binds to both DNA breaks and the Ku70/80 complex. The DNA–PK complex then attracts XRCC4 and DNA ligase IV. XRCC4, XLF, and DNA ligase IV, among others, are recruited by NHEJ for DNA end ligation repair, which is also necessary for RR in cancer cells.^294^ Compared with HR, it is quicker, does not need a homologous template, and can happen at any stage of the cell cycle. However, it is typically mutagenic.^295^, ^296^ The expression of enzymes involved in the NHEJ repair pathway rose significantly in the presence of X‐rays within 15 min of exposure, suggesting that enhanced Ku70 expression may be a significant contributor to RR in human astrocytes.^297^ Mangiferin was also discovered to increase the sensitivity of glioblastoma cells to radiation by inhibiting the NHEJ repair process by controlling the phosphorylation of many proteins, including ATM, 53BP1, and H2AX.^298^ In a separate study, it was discovered that the lncRNA LINP1 promotes DNA damage repair by reducing the amounts of caspase‐3 and poly (ADP‐ribose) polymerase (PARP) cleavage during IR, as well as by reducing the radiosensitivity of cervical cancer cells via the NHEJ pathway.^299^ These findings imply that the NHEJ repair pathway may be crucial for the regulation of the RR.

#### 6.2.1 Targeting Ku70/80 heterodimer

The NHEJ pathway, which is started by the Ku70/80 heterodimer being the first to recognize the DSB site, can also be used to repair IR‐induced DSBs.^300^ Radiation‐induced formation of Ku80 foci sensitizes cancer cells to radiation.^301^ According to one study, radiation therapy had a significant impact on the nuclear localization of Ku in patients with advanced rectal cancer.^302^ Another study discovered that radiosensitization of autophagy‐associated BECN1 abnormalities causes IR‐induced accumulation of autophagosomes, nuclear translocation, and disruption of Ku protein function, which inhibits DSB repair in malignant gliomas. When DNA is damaged, Ku may quickly connect to the damaged locations and promptly bind the frayed ends of DNA into its own pocket structure.^303^ According to clinical research, some individuals with chronic lymphocytic leukemia had B cells that were resistant to IR‐induced apoptosis. A subpopulation of radiation‐resistant cells greatly outperformed a subpopulation of radiation‐sensitive cells in terms of DNA end‐binding capacity after IR treatment.^157^, ^304^ The Ku70/80 heterodimer is a key sensor and coregulator of the NHEJ pathway for DSB repair.^104^, ^305^, ^306^ The DNA repair process was stopped when the Ku70/80 heterodimer was suppressed because DNA–PK and NHEJ activities were decreased and DNA–PKcs's affinity for DNA DSBs was dramatically diminished.^307^ Therefore, Ku has a promising future in tumor therapy. According to certain research, individuals with rectal and cervical cancer have considerably higher expression levels of Ku70 and Ku80 following chemotherapy and radiotherapy, which is linked to a poor prognosis.^302^, ^308^, ^309^ Related investigations have revealed that Ku70/80 heterodimer overexpression is directly linked to resistance to chemotherapy and radiotherapy in a variety of malignancies.^308^ For instance, shRNA‐deficient Ku70 or Ku80 causes cytotoxicity and radiosensitization in pancreatic cancer cells.^310^ Although Ku70/80 heterodimers play a very important role in the NHEJ pathway, there are inhibitors of Ku70/80 heterodimers that require further research and development.

#### 6.2.2 Targeting DNA–PKcs

Numerous studies have been done on DNA–PKcs' role in the NHEJ repair mechanism.^311^ It is now understood that DNA–PKcs recognizes DSB sites following IR damage and is recruited by Ku70/80 heterodimers to create DNA–PK complexes that eventually reconnect damaged DNA ends. These complexes include proteins like Artemis and XLF.^312^, ^313^, ^314^ Relevant NHEJ pathway elements are phosphorylated by DNA–PKcs, and ATM is also autophosphorylated‐enables DNA end processing by catalyzing the phosphorylation of Thr2609, Ser2056, and Thr2647.^315^, ^316^, ^317^ Compared with controls, cells with lower amounts of DNA–PKcs are more sensitive to infrared radiation.^318^ Due to the discovery that dsDNA can control the phosphorylation of several proteins, DNA–PKcs was first discovered.^319^ While early studies suggested that DNA–PKcs was involved in DSB repair through the NHEJ pathway, further studies have shown that DNA–PKcs also has multiple functions, including regulating the choice of HR and NHEJ repair pathways,^142^, ^320^, ^321^, ^322^ regulating cell cycle checkpoints^323^, ^324^, ^325^, ^326^ and maintaining telomeres.^326^, ^327^, ^328^ Recent research has revealed DNA–PKcs to be an essential element of the DNA damage repair process. For instance, DNA–PKcs can impact the protein stability of the cell cycle protein B1 by activating its UB via the CDH1–APC pathway.^329^ Radiation resistance is caused by c‐Myc, which influences ATM phosphorylation and DNA–PKcs kinase activity.^330^ Additionally connected to PLK1, DNA–PKcs increases cell dynamics and chromosomal segregation.^323^ After IR‐induced DDR, DNA–PKcs controls H2AX phosphorylation events in a dominant manner.^331^ Inhibition of DNA–PKcs changes several signal transduction‐related genes at the transcriptional level, ultimately impacting cell proliferation and differentiation.^332^ Up until now, radiation and genotoxic chemotherapy have both been used to treat cancer, and DNA–PKcs has been thought of as a novel target for intervention.^333^ According to studies, IR‐induced HIF‐1 (hypoxia‐inducible factor‐1) suppression effectively reversed the inhibitory impact of DNA–PKcs, decreased IR‐induced glioblastoma migration and invasion, and boosted radiation sensitivity.^334^ Human osteosarcoma cells were similarly observed to become more sensitive to IR when DNA–PKcs was inhibited.^335^ It has been suggested that targeting DNA–PKcs with a variety of inhibitors is a successful method for enhancing the responsiveness to radiotherapy and for enhancing the prognosis of cancer patients.^336^

#### 6.2.3 Targeting DNA Lig4

To connect and ligate damaged double‐stranded DNA ends, DNA Lig4 is often recruited to DNA damage sites along with XRCC4 and XLF. DNA Lig4 is an important DNA repair component of the radiation‐induced NHEJ repair process.^107^, ^337^ Lig4 deficiency causes a rare primary immunodeficiency known as Lig4 syndrome.^338^ Lig4 is thought to be significant in DDR because patients with Lig4 syndrome are more susceptible to radiation, as well as more likely to experience neurological problems, bone marrow failure, and malignancy.^339^ The starvation of numerous other proteins, such as XRCC4, which is essential for stabilizing Lig4, regulates Lig4 activity, but several additional coregulated variables also affect Lig4 stability.^340^ Additionally, DNA‐binding protein 1 can inhibit the production of Lig4 to adversely affect the DNA repair process^341^. Additionally, Lig4 is a substrate for DNA–PK, and the human Lig4 Thr650 site has a DNA–PKcs phosphorylation site.^342^ Lig4 affects the RR process through the above molecular mechanisms. Lig4 inhibitor medication research holds great promise for cancer treatment. Some studies have discovered that by selectively targeting Lig4, rabeprazole and U73122 can disrupt the adenylate transfer step, DNA re‐linking, and hinder the repair of DNA damage caused by IR.^187^ According to a different study, SCR7 prevents Lig4 from mediating DNA binding by interfering with its attachment.^188^ On the other hand, the radiosensitivity of wild‐type Lig4 mouse embryonic fibroblasts is compromised by the drug NU7026.^343^ Although Lig4 inhibitors offer a lot of potential as cancer treatments, it is possible that they will not work against mutant cancer cells, which would compromise the effectiveness of radiotherapy. Therefore, more in‐depth studies on the role of Lig4 in radiation‐induced DDR are needed in the future.

### 6.3 Other therapeutic targets

MDC1, a sizable modular phosphorylated protein scaffold, is crucial to the DDR procedure.^344^, ^345^ Through the interaction of its BRCT structural domain with the H2AX tail, MDC1 is anchored to the damaged location after DSB formation. Additionally, 53BP1 typically travels toward the lesion site under the control of MDC1, demonstrating how MDC1 and 53BP1 typically work together.^346^ In turn, 53BP1's role in the DDR is dependent on its ability to recognize methylated histone H4 (H4K20me2) and ubiquitinated histone H2A (H2AK15ub) through its tandem Tudor structural domain^347^ Following radiotherapy, Ku70, gamma‐H2AX, and MDC1 colocalized in nuclear foci in colon cancer cells, according to an immunoprecipitation study.^348^ According to reports, MDC1 can be controlled by Bora, which can then be phosphorylated by MDC1 to prevent the development of MDC1 foci caused by irradiation. Downregulating Bora also increases resistance to IR, which may be caused by an increase in DSB repair.^349^ It has been demonstrated that radiation therapy for head and neck cancer patients with low expression of 53BP1 leads to more complete responses and increased survival times compared with radiation therapy for patients with high expression of 53BP1. In general, IR‐induced DNA damage attracts MDC1 to the site of damage within about a minute of irradiation, providing an H2AX‐dependent interaction platform for the recruitment of additional DNA damage repair proteins.^350^

One important regulator of DNA damage repair, namely DSB repair, is PARP1.^196^, ^351^, ^352^ The earliest reaction of DDR is the poly (ADP‐ribosyl) ation (PARylation) of proteins.^353^ A crucial signaling sensor for DDR, PARP1 is in charge of preserving the stability and integrity of the genome.^354^ Post‐translational PARylation of proteins is thought to represent a local indication of DNA damage since they include poly‐binding domains, and DDR factors can control the activity of related proteins.^355^ Since it has been shown that inhibiting PARP1 makes cancer cells more responsive to radiotherapy, PARP1 inhibitors are being created.^356^, ^357^ PARPi was found to be sensitive to conventionally treated cervical cancer cells and could enhance the efficacy of chemotherapy, radiotherapy and thermotherapy.^358^ PARPi work mainly by inhibiting the catalytic activity of PARP1 and by limiting PARP1, both of which are designed to inhibit PARylation or inhibit the release of PARP1.^194^, ^359^ In addition to inhibiting PARP1's ability to repair DSBs, PARPi also attracts other proteins to DSB sites, which exacerbate a drug's cytotoxic effects by impeding DNA replication. Because of this, cells need functional HR to get over these obstacles and resume cell cycle progression; otherwise, PARPi causes cell death in tumor cells that lack HR.^360^ For instance, Olaparib was the first PARP inhibitor that the United States Food and Drug Administration has approved for treatment in individuals with ovarian cancer and BRCA mutations. Inhibiting PARP1 with Olaparib prevented the recruitment of ALC1 to the DNA damage site, which prevented the removal of nucleosomes, which prevented the recruitment of RPA2 and RAD51, and ultimately prevented HR repair in hepatocellular carcinoma.^361^ In addition to promoting the accumulation of DSBs, cell cycle blockade, and apoptotic cell death, the additional PARPi niraparib also inhibits the DDR and significantly slows the growth of multiple myeloma (MM) xenografts in nude mice. Inhibition of PARP1 increases the sensitivity of genotoxic drugs and is an important avenue for the treatment of MM. This study will help increase the use of PARP1 inhibitors in combination with additional chemotherapeutic medicines, enhancing the therapeutic efficacy of radiotherapy and decreasing the likelihood that PARP1 inhibitors may become resistant to drugs.^196^ PARP1 inhibitors have been used successfully in clinical cancer therapy for decades, but the molecular mechanisms involved remain to be elucidated. Currently, PARPi resistance is an important issue for clinical application, and the mechanisms of PARPi resistance are mainly of the following four categories: (I) affecting the cellular availability of inhibitors; (II) reactivating HR, (III) directly influencing the quantity and activity of PAR chains, and (IV) influencing replication fork protection.^360^ The mechanisms behind the radiosensitizing effects of PARPi need to be further explored and understood, even though they have been discovered, tested in clinical trials, and shown to increase cancer sensitivity to radiation.

H2AX is a core histone H2A variant that is rapidly phosphorylated at the S139 location in response to DSBs, resulting in the formation of H2AX foci.^362^ In an earlier report, γH2AX was still present on re‐exposure to IR after treatment with a variety of radiosensitizing drugs, suggesting that the sensor could be used for cancer monitoring. Nowadays, γH2AX localization at DNA damage sites can be observed using γH2AX monoclonal antibodies and immunofluorescence hybridization^363^, ^364^; γH2AX foci can be observed even at very low doses of IR irradiation, but the foci disappear as the DNA damage is repaired, and their disappearance usually occurs earlier than that of other IR‐exposed reactive proteins.^365^, ^366^ Therefore, it has been suggested that the γH2AX locus may represent a 1:1 DSB and can be used as a biomarker of DNA damage.^367^ Additionally, H2AX can serve as a platform for the recruitment of additional DNA repair proteins like BRCA1,^368^ 53BP1,^369^ MDC1^370^ RAD51,^371^ and so on. γH2AX is currently frequently employed in immunofluorescence labeling or immunocytochemistry of lesions because it is believed to be more consistent with the traits of DNA damage sensors than the expression of DSB repair genes.^372^, ^373^, ^374^, ^375^

The genetic switches HIF‐1 and HIF‐1, which control cellular sensing and reaction to changes in oxygen status, make up HIF‐1.^376^, ^377^ Hypoxia induced by vascular injury under radiotherapy conditions and reactive oxygen species produced by insulin can activate HIF‐1, and radiation exposure can promote the translation of HIF‐1α.^378^, ^379^ It has been shown that in radioresistant NSCLC cells, post‐IR PAI‐1 mediates radioresistance in neighboring cells through paracrine secretion by upregulating HIF‐1 secretion.^380^ Radiation therapy (IR) triggers a few signaling pathways that have an impact on HIF‐1 expression when metabolic alterations take place in radioresistant cancer cells.^381^ In tumor stem cells, mitochondria‐mediated RR may be mediated by HIF‐1.^382^ In conclusion, HIF‐1 may enhance RR and boost the effectiveness of radiation.

HDACs are enzymes that catalyze the removal of acetyl groups from lysine residues at the amino terminus of histones.^383^, ^384^, ^385^ Previous correlative investigations have demonstrated the watchdog function of HDACs in IR‐induced DNA damage and their promotion of RR. For instance, after radiotherapy, HDAC6 suppression causes apoptosis in glioblastoma cells.^386^ HDAC controls the acetylation of proteins involved in DSB repair as well as the acetylation of histones and nonhistone proteins.^387^ Over the past few years, a variety of HDAC inhibitors have been created, which can suppress HDAC to improve RR. HDAC inhibitors suppress many proteins crucial for DNA DSB repair by downregulating key proteins in the HR and NHEJ repair pathways, according to in vitro investigations.^388^

A cell cycle regulator called CDK1 controls the advancement of G1 and the G1/S transitions as well as mitosis, which promotes the G2/M phase transition.^389^ When IR causes cellular DNA damage, CDK1 is deactivated and cell cycle progression is stopped at the G2 checkpoint, boosting DSB repair.^390^ Cancer cells promote CDK1 activity, a feature of great interest for targeted cancer therapy. This is based on the discovery that S phase cells inhibit DNA synthesis via the ATM/NBS1 and ATM/CHK2/CDC25A/CDK2 pathways when exposed to radiation.^391^, ^392^ Therefore, inhibiting CDK1 and thereby controlling cell cycle progression may be a potential target for radiotherapy.^393^, ^394^

Wee1 controls the cell cycle's entry into the mitotic G2 to M transition negatively. Wee1 can phosphorylate the CDK1 Tyr15 site. Wee1 can also prevent the cell cycle protein B1/CDK1 complex from moving from the cytoplasm to the nucleus before the start of mitosis, and it can phosphorylate the cell cycle protein B1/CDK1 complex to inactivate it.^395^, ^396^, ^397^ The activity of Wee1 is elevated in the S/G2 phase and then decreases after hyperphosphorylation in the M phase.^398^, ^399^, ^400^, ^401^ Inhibiting Wee1 activity may be a strategy for cancer radiation because Wee1 has been identified as a potential target for DSB repair.^402^, ^403^, ^404^

CHK1 is a crucial regulator that encourages cell cycle arrest and activation of DNA repair via cell cycle checkpoints, much as Wee1 and CDK1.^405^ By interacting with RAD51, which encourages S‐phase and G2/M cell cycle checkpoints and is important in the control of replicative stress in cells, CHK1 is activated in the HR repair pathway.^406^ Studies have revealed that CHK1 has a major impact on cell survival and proliferation, making it a viable target for radiation sensitization of cancer cells.^407^ Recently, several CHK1 inhibitors have been created, including LY2606368,^245^, ^408^, ^409^ PF‐00477736,^410^, ^411^ and SRA737.^412^, ^413^, ^414^ These medications have been shown in studies to inhibit CHK1, which reduces DNA synthesis and raises H2AX levels, accumulating DNA damage and making cancer cells more sensitive to radiotherapy. The study also discovered that other DNA‐damaging medications can be used with CHK1 inhibitors in cancer treatment.^415^, ^416^, ^417^, ^418^ In the case of condylomatous lymphoma and diffuse large B‐cell lymphoma cancer cells, the CHK1 inhibitor AZD6738 and the Wee1 inhibitor AZD1775 delay S‐phase and accumulate DNA damage, and the synergistic impact of cytotoxicity is extremely apparent.^417^

Checkpoint kinase 2 (CHK2) is a vital component of the DDR and is crucial in regulating DSB‐induced cell cycle arrest and death. Activated CHK2 can phosphorylate associated proteins involved in DNA damage repair, cell cycle control, and apoptosis, such as P53 and BRCA1, for damage signaling. CHK2 is activated by ATM phosphorylation when a DSB is created.^419^ When CHK2 is defective, it increases the risk of tumorigenesis through replication‐induced genotoxicity.^420^ Therefore, CHK2 is a prospective target for cancer therapy. AZD7762, a CHK1/CHK2 inhibitor,^250^ honokiol or tunicamycin are less cytotoxic to NSCLC cells, but can cause accumulation of DSB damage, arrest cells in G2/M phase and induce apoptosis, which may enhance radiosensitization of NSCLC cells exposed to carbon ions rather than X‐rays.^248^ Breast cancer cells undergo G2/M phase arrest and apoptosis as a result of timosaponin A III (TAIII), which causes DNA damage, activates the ATM/CHK2 pathway, increases p‐H2AX and p‐p38 levels, and causes cell cycle arrest.^421^

**FIGURE 9:**
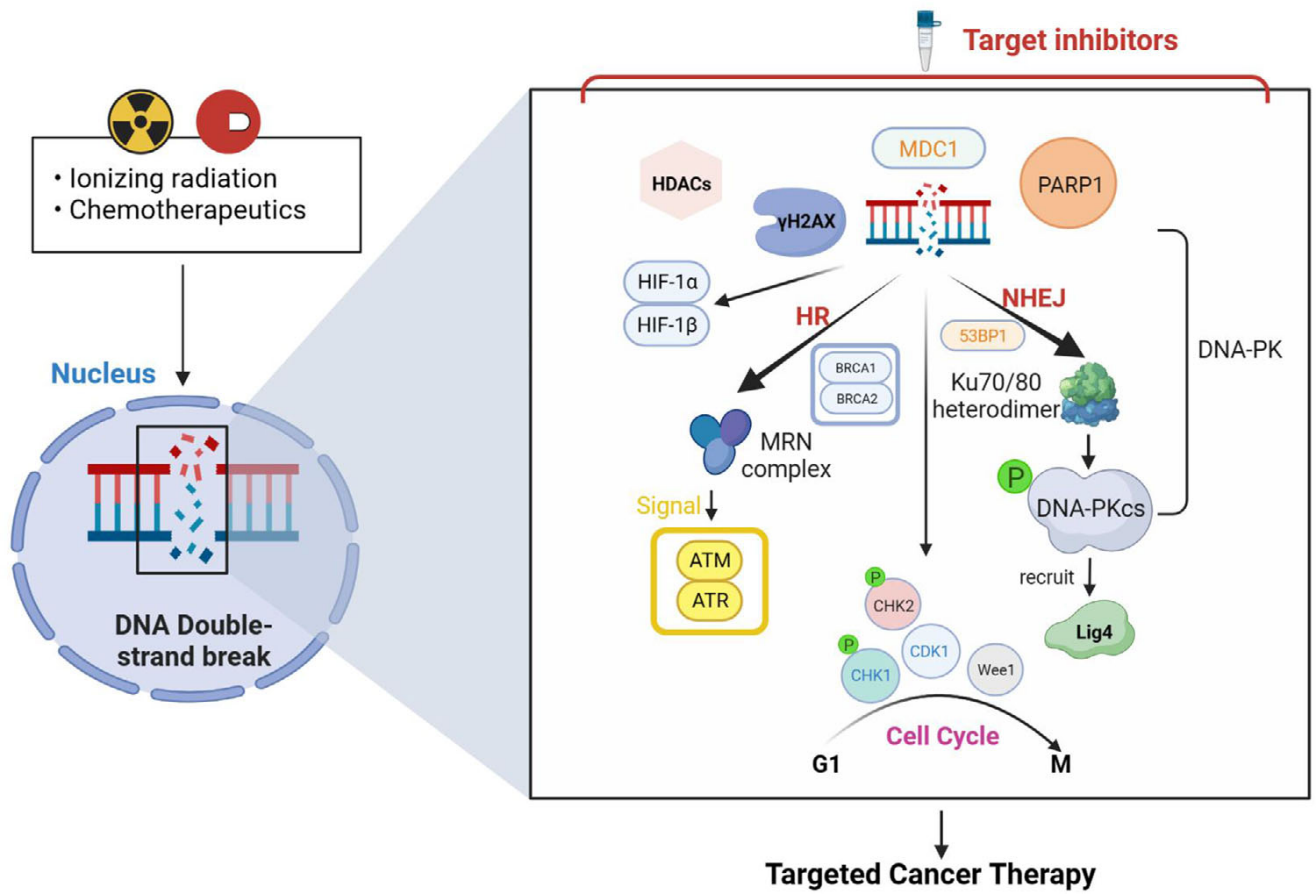
HR and NHEJ are the two primary repair procedures in the DSB damage repair pathway. DNA double‐strand breaks can be harmed by radiation or toxins. Pathway proteins that are important in DSB damage repair include the MRN complex, BRCA1/2, ATM/ATR, Ku70/80 heterodimer, DNA–PKcs, Lig4, MDC1, 53BP1, HDACs, HIF‐1, PARP1, and H2AX. The creation of inhibitors to enable tailored therapy for the relevant tumors might conceivably use these proteins as targets.

The clarification of the structure and function of important genes and proteins in the DDR signaling network, as well as a greater comprehension of the molecular mechanism of IR‐induced DSBs, are prerequisites for the clinical discovery of further potential therapeutic targets in the future. The involvement of genes and proteins in the control of DNA damage repair in radiosensitization is the subject of numerous recent studies, which will aid in the creation of more focused and specialized medications for use in the treatment of cancer. To develop safer, more dependable, and more effective inhibitors and improve radiotherapy, it will be necessary in the future to have a deeper understanding of the molecular mechanism of IR‐induced DSBs and how inhibitors are engaged in radiation‐induced DNA damage (Figure 9).

## 7 DISCUSSION AND PROSPECTS

The MRN complex, the Ku70/80 heterodimer, and their interactions in controlling DNA DSBs repair and pathway choice are briefly discussed in this article. MRE11, RAD50, and NBS1 make up the protein complex known as the MRN complex. In the early stages of DNA DSB damage, it can detect and enrich DSBs. Next, it can excise the broken DNA ends with associated nucleases to create ssDNA at the 3′ end and start HR repair. ssDNA‐binding protein replicating protein A (RPA) can coat the 3′‐ended ssDNA, and RAD51 is subsequently swapped with SSPA‐coated ssDNA to create RAD51 nuclear filaments. After establishing a D‐ring and synthesizing DNA along the template, the newly created ssDNA–RAD51 nuclear filament enters the sister chromatids and completes HR repair. The NHEJ repair pathway can be started by the Ku70/80 heterodimer, a circular protein complex made up of the Ku70 and Ku80 monomers that can first detect DSB sites and bind to start it. The Artemis nuclease and DNA‐dependent protein kinase catalytic subunits (DNA–PKcs) were attracted to DSB locations. To finish NHEJ repair, DNA ligase IV (Lig4), XRCC4, and XLF are called upon after DNA–PKcs has been activated. To control and choose distinct repair routes (HR or NHEJ) for DNA DSB damage repair, the MRN complex and the Ku70/80 heterodimer can both coexist and competitively bind at the same DSB site.

**FIGURE 10:**
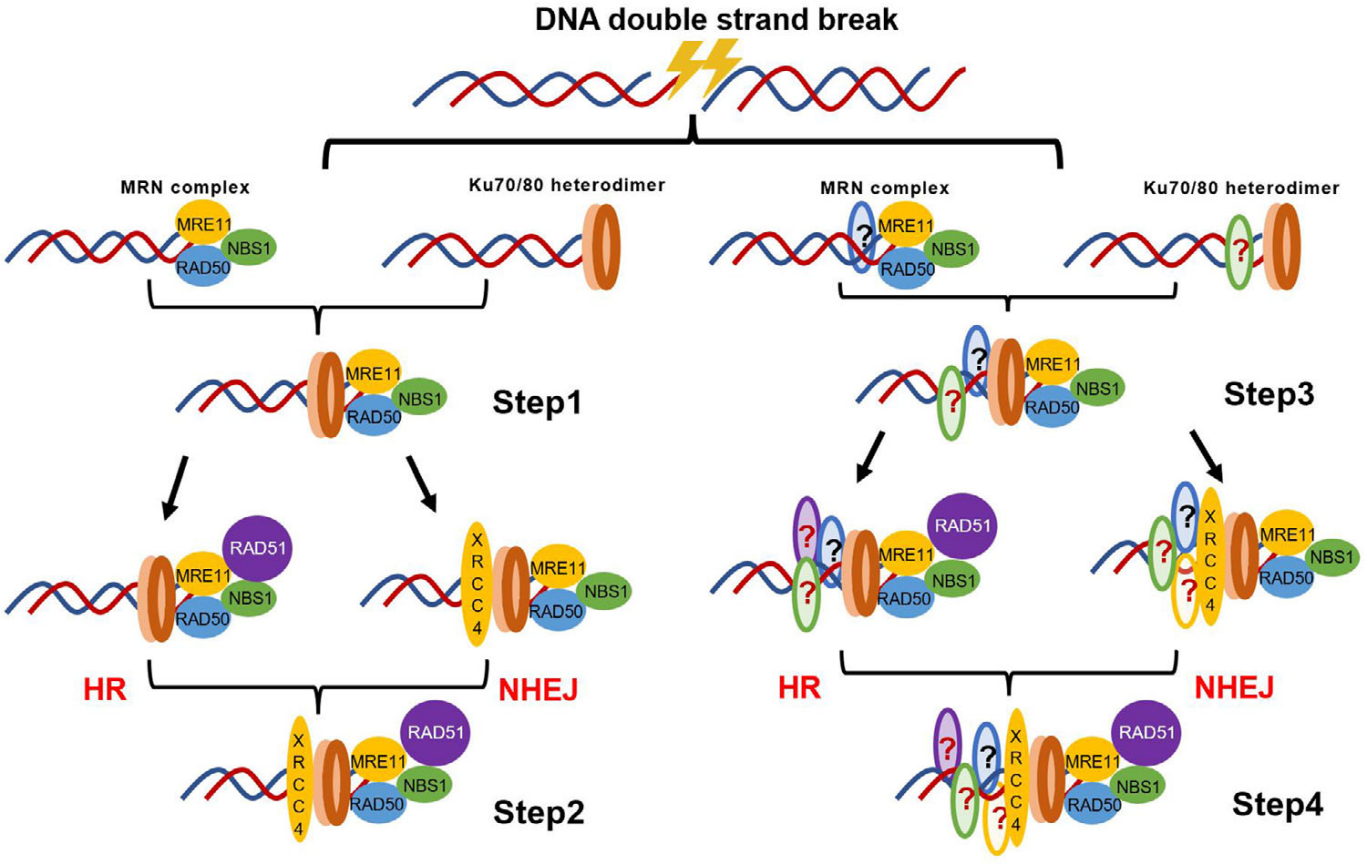
Diagram illustrating the pattern used in the research of the MRN complex and the Ku70/80 heterodimer's bias in the selection of DNA double‐strand break repair pathways using biochemical and bioinformatics techniques. Step 1: Look for cobinding sites at DSBs for MRN complexes containing Ku70/80 heterodimers. Step 2: The ratio of HR to NHEJ incidence can be determined by comparing the cobinding regions at DSBs of the MRN complex and Ku70/80 heterodimer with the binding regions of RAD51 and XRCC4. Step 3: Finding relevant regulatory proteins close to the MRN complex and the Ku70/80 heterodimer's cobinding area. Step 4: Evaluate and compare the protein sets close to the cobinding region, the RAD51‐binding DSB region, and the XRCC4‐binding DSB region to determine the critical elements governing the bias of the cobinding DSB repair pathway.

We think that the combination of bioinformatics and biochemical methods, such as End‐seq, ChIP‐Seq, ChIP‐MS, Re‐ChIP‐Seq, Re‐ChIP‐MS, and PICh‐MS, can contribute to the comprehension of DSB repair pathway choice and provide an answer to the question of how MRN complexes collaborate with Ku70/80 heterodimers at the same DSB site. For instance, we will be able to examine how MRN complexes and Ku70/80 heterodimers bind to the same DSB site and the percentage of each that binds to the same DSB site using high‐throughput sequencing techniques like ChIP‐Seq. The choice bias of DNA DSB repair pathways was then ascertained by combining the binding ratios of RAD51 and XRCC4, the crucial proteins that function downstream of the HR and NHEJ repair pathways, at the identical DSB site. Additionally, the MRN complex's interaction with the Ku70/80 heterodimer at DSB sites and the decision to employ the HR or NHEJ repair pathways were identified using the PICh (reverse ChIP assay) and mass spectrometry techniques (Figure 10).

Understanding the structure, functionality, and synergistic competition between Ku70/80 heterodimers and MRN complexes in the selection of DNA damage repair pathways has advanced greatly. For the HR and NHEJ processes, MRN complexes and Ku70/80 heterodimers serve as DSB sensors and initiation proteins. Further research and discussion will be conducted to determine how different DSB kinds, gene types, and cell cycles affect the binding and retention of MRN or Ku70/80 heterodimers at DSBs and the selection of DNA DSB damage repair pathways.

The greatest hazardous harm to cells is known to be caused by DNA DSBs. Each essential protein has a regulatory function that is crucial to the repair of DNA DSBs. These proteins are essential for signal transduction or for controlling one another. The critical proteins at each node of the DNA damage signaling network can theoretically serve as therapeutic targets for illnesses associated with them. The development of numerous protein signaling molecule inhibitors for clinical trials has resulted from increased research into relevant therapeutic targets, but the underlying molecular mechanisms are still poorly understood. In particular, there are still several significant scientific questions in the investigation of the IR‐induced DNA damage repair pathway that need to be answered. For instance, in the IR‐induced DNA damage signaling network, the substrates of several phosphorylation events and the accompanying molecular pathways are not entirely understood. Unanswered questions include how these phosphorylation events take place, what additional signaling molecules are controlled during the process, how this influences the selection of ensuing repair pathways, and so on. But with the further advancement of science and technology, like as mass spectrometry and high‐throughput sequencing, we think it will be possible to completely understand and respond to the problems raised above.

Recent research has demonstrated that the use of radiotherapy and immunotherapy together can cause a variety of biological reactions, including the induction of cell death. Therefore, the modulation of radiation‐associated signaling and immunotherapy signaling should be considered when creating inhibitors that can enhance resistance to radioimmunotherapy.^422^ Additionally, there must be enough information to evaluate an inhibitor's potential for repairing DNA damage as well as its safety and toxicity in normal tissues, which are relevant therapeutic targets. Studies on the use of combinations of radiotherapy and chemotherapy are crucial, taking care to minimize harm from overlapping toxicity. This is because we need to provide patients with radiotherapy that is more targeted, less toxic, more efficient, and with a more trustworthy safety profile. Last but not least, we also require enough early sensitive biomarkers for RR prediction, prevention, and control. The investigation of various DNA damage sensors and DNA damage repair regulatory proteins as potential targets to increase radiotherapy's sensitivity is still ongoing, but the future emphasis should be on how to apply this basic research in the clinic. All things considered, research into DNA DSB damage repair signaling pathways will significantly aid in the emergence and creation of tailored treatments for associated illnesses, enhance the efficacy of radiation, and enhance the effectiveness of cancer treatment.

## AUTHOR CONTRIBUTIONS

S. G., P .Z., and J. T. conceived the project and wrote the manuscript. X. S., H. Z., and H. G. provided technical support. All authors have read and approved the final manuscript.

## CONFLICT OF INTEREST STATEMENT

All authors declared no conflict of interest.

## ETHICS STATEMENT

Not applicable.

## Data Availability

All data are available from the corresponding authors upon request.
